# Application of quantitative proteomics to discover biomarkers for tick resistance in cattle

**DOI:** 10.3389/fimmu.2023.1091066

**Published:** 2023-01-30

**Authors:** Ali Raza, Benjamin L. Schulz, Amanda Nouwens, Muhammad Noman Naseem, Muhammad Kamran, Emily F. Mantilla Valdivieso, Edward D. Kerr, Constantin Constantinoiu, Nicholas N. Jonsson, Peter James, Ala E. Tabor

**Affiliations:** ^1^ Queensland Alliance for Agriculture & Food Innovation, Centre for Animal Science, The University of Queensland, St Lucia, QLD, Australia; ^2^ The University of Queensland, School of Chemistry and Molecular Biosciences, St. Lucia, QLD, Australia; ^3^ College of Public Health, Medical & Veterinary Sciences, James Cook University, Townsville, QLD, Australia; ^4^ Institute of Biodiversity Animal Health and Comparative Medicine, University of Glasgow, Glasgow, United Kingdom

**Keywords:** proteomics, biomarker discovery, immune response, skin integrity, host resistance, wound healing, *Rhipicephalus australis*

## Abstract

**Introduction:**

Breeding for tick resistance is a sustainable alternative to control cattle ticks due to widespread resistance to acaricidal drugs and the lack of a protective vaccine. The most accurate method used to characterise the phenotype for tick resistance in field studies is the standard tick count, but this is labour-intensive and can be hazardous to the operator. Efficient genetic selection requires reliable phenotyping or biomarker(s) for accurately identifying tick-resistant cattle. Although breed-specific genes associated with tick resistance have been identified, the mechanisms behind tick resistance have not yet been fully characterised.

**Methods:**

This study applied quantitative proteomics to examine the differential abundance of serum and skin proteins using samples from naïve tick-resistant and -susceptible Brangus cattle at two-time points following tick exposure. The proteins were digested into peptides, followed by identification and quantification using sequential window acquisition of all theoretical fragment ion mass spectrometry.

**Results:**

Resistant naïve cattle had a suite of proteins associated with immune response, blood coagulation and wound healing that were significantly (adjusted P < 10- 5) more abundant compared with susceptible naïve cattle. These proteins included complement factors (C3, C4, C4a), alpha-1-acid glycoprotein (AGP), beta-2-glycoprotein-1, keratins (KRT1 & KRT3) and fibrinogens (alpha & beta). The mass spectrometry findings were validated by identifying differences in the relative abundance of selected serum proteins with ELISA. The proteins showing a significantly different abundance in resistant cattle following early and prolonged tick exposures (compared to resistant naïve) were associated with immune response, blood coagulation, homeostasis, and wound healing. In contrast, susceptible cattle developed some of these responses only after prolonged tick exposure.

**Discussion:**

Resistant cattle were able to transmigrate immune-response related proteins towards the tick bite sites, which may prevent tick feeding. Significantly differentially abundant proteins identified in this research in resistant naïve cattle may provide a rapid and efficient protective response to tick infestation. Physical barrier (skin integrity and wound healing) mechanisms and systemic immune responses were key contributors to resistance. Immune response-related proteins such as C4, C4a, AGP and CGN1 (naïve samples), CD14, GC and AGP (post-infestation) should be further investigated as potential biomarkers for tick resistance.

## 1 Introduction

The Australian cattle tick, *Rhipicephalus australis*, belongs to one of the five clades of *R. microplus* species complex and causes significant financial losses to the cattle industry exceeding AUD$150 million annually (1). Globally, ticks and tick-borne diseases affect 80% of the cattle population, causing financial losses of US$ 22-30 billion annually (2). The severe economic losses of tick infestations on cattle production necessitate the development of effective control strategies to combat tick infestations. Tick control has heavily relied on the use of acaricidal drugs. However, total dependence on acaricides is not a sustainable strategy due to the development of resistance and economic, environmental and consumer concerns (3, 4). The differences in tick burdens of cattle across different breeds under the same environmental conditions have been associated with host resistance. For example, *Bos indicus* breeds are usually more resistant than *Bos taurus* breeds. Tick resistance is generally manifested against larval stages resulting in the death of the larvae within 24 hours after infestation, also known as larval rejection (5). Therefore, using the host’s natural resistance to ticks could provide an opportunity to develop an alternative tick control strategy.

Previous studies investigated the local (skin) and systemic (blood) mechanisms of tick resistance using genomic, transcriptomic and immunological approaches with the hope of identifying biomarkers for tick resistance. For example, Piper et al. (6) reported that extracellular matrix and collagen encoding genes were upregulated in the skin of Brahman as compared to Holstein-Friesian cattle, potentially indicating differences in ease of tick-feeding and development of feeding lesions. Such findings suggest a potential role of physical attributes of the skin, including efficient capacity for wound healing in tick resistance. Several other studies have also documented differences in gene expression levels and cellular responses in the skin of tick-resistant and susceptible cattle (7–9), however, until recently, only one study has focused on skin proteins. The authors suggested that the epidermal permeability barrier of the skin may be a critical component of tick resistance in cattle (10).

In addition, studies have provided insights into the immunological factors associated with tick resistance in various cattle breeds (11–13). Both innate and acquired immune responses appear to be involved in the host response to tick infestation. Previously, Wagland (14) suggested that resistance to tick infestation is acquired rather than being innate. However, the importance of innate response was evident from the considerably shorter period for which the Brahman (*Bos indicu*s) cattle remained susceptible to tick infestation compared to Shorthorn (*Bos taurus*) cattle (reviewed by 15). This was supported by our recent findings reporting a higher abundance of immune-related proteins in tick-resistant Santa Gertrudis cattle as compared to susceptible cattle before any tick exposure (16). On the other hand, susceptible animals have been shown to develop a persistent innate inflammatory response to tick infestation (6, 17–19), possibly facilitating tick feeding. Similarly, a role for acquired immunity in tick resistance has been evidenced by variation in cell-mediated responses and hypersensitivity reactions at tick bite sites in indicine and taurine cattle (17, 20, 21).

Most of the previous studies focused on investigating local and systemic host responses to tick infestation through the characterization of immune responses (cellular and humoral) and variations in gene expression (RT-PCR and microarray) or identifying quantitative trait loci in genome-wide analyses (7, 11, 18, 22, 23). Recently, Moré et al. (24) identified more than a hundred immune-related genes encoding cytokines, chemokines, CD markers, acute phase proteins, complement proteins, integrins, and transcription factors using a high throughput RNA sequencing technology to compare gene expression in tick-resistant and susceptible Braford cattle. Whereas Mantilla Valdivieso et al. (25) identified genes related to immune, tissue remodelling, and angiogenesis functions from tick-resistant and susceptible Brangus composite breed cattle. In response to tick infestation, transcriptome analyses provide a broader picture of functional activity at the cellular level by summarizing coding and non-coding transcriptional activity and gene expression. However, the proteome is the functional system of the genome by which the cells react to environmental signals (26), thus proteomics is important to understanding cellular biology and host mechanisms to tick infestation. Recently, we reported that sequential window acquisition of all theoretical ions mass spectrometry (SWATH-MS) is capable of identifying variation in abundances of proteins in serum samples from Santa Gertrudis cattle and concluded that such differences could be used to identify potential biomarkers for tick resistance (16). However, these findings require further validation in other breeds of cattle and wider environmental conditions. It is also important to investigate the changes in skin proteomes representing local responses to the parasite. Based on the previous findings, we hypothesized that tick-resistant and susceptible individuals from other cattle breeds also develop different local and systemic responses which can be detected at the protein level. Therefore, in this study, we used SWATH-MS with serum and skin samples from Brangus cattle (3/8 Brahman and 5/8 Angus) to reveal the changes in proteomes of tick-resistant and -susceptible cattle, subsequently exploring the systemic and local host response to tick infestation.

## 2 Materials and methods

### 2.1 Animals

Thirty Brangus steers aged 6-8 months with an average weight of 200 (± 9.8) kg were sourced from a tick-free region (Morven, Queensland) in Australia. The animals had no previous exposure to cattle ticks and were vaccinated for tick fever pathogens (*Babesia bovis*, *Babesia bigemina* and *Anaplasma marginale*) (Combvac 3 in 1^®^ sourced from Tick Fever Centre, Department of Agriculture and Fisheries, Biosecurity Queensland) at the farm four weeks before they were transported to the University of Queensland’s Pinjarra Hills Research Facility. The study was conducted with approval from Animal Ethics Unit, the Office of Research Ethics, The University of Queensland (Animal ethic approval No. QAAFI/469/18).

### 2.2 Tick infestation and animal phenotyping

**Figure 1:**
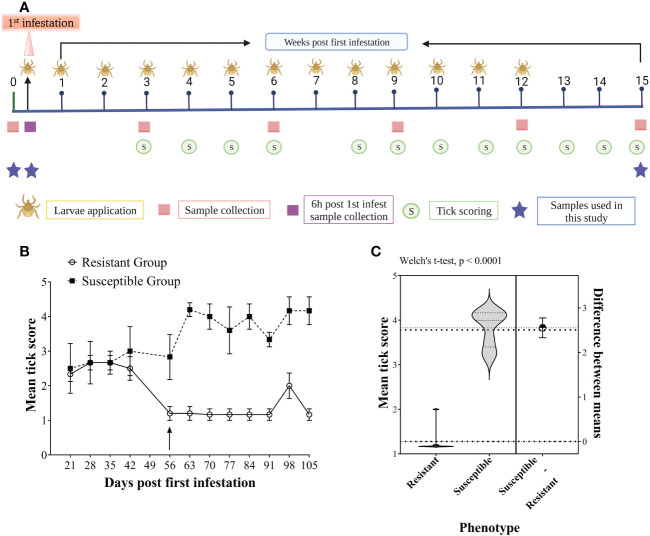
Experimental plan of the study **(A)**, representing the study design timeline of the artificial tick infestations showing the frequency of larvae applications (larvae), tick scoring (circles), sampling (squares) and selected sampling timepoints for this study (stars). Tick scoring for host resistance phenotyping of Brangus steers, **(B)** Weekly group mean tick scores for the six most susceptible (dotted line, squares) and six most resistant (solid line, circles) animals measured from 21 days to 105 days post-first infestation. The arrow shows the time at which a clear separation of the two groups was evident from the mean tick scores. The values are presented as the group mean ± SEM. **(C)** Violin plots showing the comparison of mean tick score (week 8-15) values between resistant and susceptible host phenotype compared with Welch’s t-test (P < 0.0001).

For animal phenotyping based on tick burden, an intensive artificial tick infestation trial was carried out using larvae of a Non-Resistant Field Strain of *R. australis*, as reported by (25). Briefly, 30 animals were artificially infested with 10 000 (0.5 g) *R. australis* larvae applied weekly on the back of each animal for 12 consecutive weeks with a total of 13 infestations, along with concurrent exposure to the natural tick infestation on pastures. Tick burdens for each animal were determined by undertaking weekly tick counts for 13 weeks, from week 3 to 15, except week 7, when scoring could not be performed due to staff unavailability. The experimental plan is presented in **Figure 1A**. Briefly, tick burden was estimated by the number of unfed/semi/fully engorged adult female ticks by observing the rear (back legs between the tail base and the genitals) and feeling by rubbing the palm of a hand on the back, belly, dewlap and lateral sides of the body while the animal was restrained in a cattle crush. A scoring scale from 1 to 5 (1 = 0-50, 2 = 50-100, 3 = 100-200, 4 = 200-300, and 5 = >300 ticks) was used to record tick burden weekly. The scores correspond to the estimated number of female adult ticks (unfed/semi/fully engorged) found on one side of the animal’s body. The first seven weeks following the first infestation were considered the adaptation period. Animals were divided into different tick phenotypes based on the mean tick scores from weeks 8 to 15. The animals with the highest mean tick scores (>3.5) were classified as “susceptible (n = 6)”; the animals with the lowest mean tick score (<1.5) representing the least tick burden were classified as “resistant (n = 6)”, and the rest of the animals were classified as “median”. The tick scores for the susceptible and resistant groups were compared using Welch’s t-test with GraphPad Prism (version 9).

### 2.3 Sample collection

Samples were collected at time points including day 0 before tick infestation (naïve animals), 6 hours post-first infestation (to study early host responses to tick infestation), and then after every three weeks from the first infestation until the end of the trial (**Figure 1A**). The cattle were restrained in a crush and blood samples were obtained *via* jugular venipuncture into 9 mL Vacuette^®^ Z clot activator tubes at each time point and serum was collected and stored at -20°C for further use. Skin samples were collected from the perineal area of the cattle using an epidural injection of 3 mL of lignocaine HCl (20 mg/mL) (Troy Laboratories Pty. Limited, Sydney, Australia) to desensitize the perineum. Skin biopsies were collected with 8-mm biopsy punches (Meister Surgical Instrument^s®^, Sialkot, Pakistan), snap-frozen in liquid nitrogen, transported in ethanol-dry ice slurry and stored at -80°C until further use. Blood and skin samples collected from unexposed animals (referred to as “naïve resistant and “naïve susceptible”); 6 hours post-first infestation (6h PFI) and at the end of tick infestation trial, 105 days PFI (week 15), (referred to as “resistant and “susceptible”) were used in this study. Samples from the two extreme groups (resistant and susceptible) were included for quantitative proteomics analysis.

### 2.4 Protein extraction and sample preparation

Frozen skin biopsies were homogenized in lysis buffer comprised of T-PER™ Tissue Protein Extraction Reagent (ThermoFisher Scientific^®^, USA) and Halt™ Protease Inhibitor Cocktail (100X) (ThermoFisher Scientific^®^, USA). Briefly, each biopsy was sliced into thin pieces with a surgical blade (No. 10) and homogenized in 1 mL of lysis buffer with zirconia (1mm and 2 mm) and steel (3 mm) beads (Miniature Bearings, Australia) using a tissue lyser (ThermoFisher Scientific, USA) in 2 mL screw cap tubes. The samples were homogenized for two cycles of two minutes each at 30 Hz/Sec, with incubation on ice for two minutes after each cycle, followed by centrifugation at 16,000 x g for 1 hour at 4°C. The supernatant was collected and stored at -20°C for further use. Protein concentration in serum samples and skin extracts was quantified by Qubit protein assay (Thermo Fisher Scientific^®^, USA).

Samples were prepared for mass spectrometry in randomized blocks, for each sample (skin and serum), 100 µg of total protein was denatured (100 µL of 8 M urea, 50 mM ammonium bicarbonate (ABC)), reduced (5 mM DL-Dithiothreitol), and alkylated (25 mM final concentration of iodoacetamide) using Pierce concentrator 10K molecular weight cut-off columns (Thermo Fisher Scientific^®^, USA) as described previously (16). Following alkylation, proteins were digested with 4 µg trypsin in 50 mM ABC (Trypsin Gold Mass-Spec Grade, Promega^®^) by overnight incubation at 37°C in a thermomixer with shaking at 400 rpm. The digested peptides were desalted with C18 ZipTips (Millipore^®^, USA) following the manufacturer’s instructions. For skin and serum samples, a separate pooled sample generated by taking 3 µL from each sample (before desalting), totaling approximately 108 µg peptides was subjected to fractionation using Pierce High pH Reversed-phase Peptide Fractionation kit (Thermo Fisher Scientific^®^, USA). Peptides eluted in eight separate fractions of acetonitrile (300 µL for each 5%, 7.5%, 10%, 12.5% 15%, 17.5%, 20% and 50%) in triethylamine (0.1%) were lyophilized and resuspended in 0.1% trifluoroacetic acid.

### 2.5 Mass spectrometry

Samples were analyzed in a random order, and peptides were measured by liquid Chromatography with tandem mass spectrometry (LC-MS/MS) experiment using a data-independent acquisition (DIA) protocol with a Shimadzu^®^ Prominence nanoLC system along with a TripleTOF 5600 mass spectrometer with a Nanospray III interface (SCIEX^®^) as described previously (27). Based on ZipTip binding capacity, approximately 2 µg of peptides were desalted on an Agilent C18 trap (pore size 300 Å, particle size 5 μm, 0.3 mm i.d. × 5 mm) at a flow rate of 30 µL/min for 3 min, followed by separation on a Vydac EVEREST reverse-phased C18 HPLC column (pore size 300 Å, particle size 5 μm, 150 μm i.d. × 150 mm) at a flow rate of 1 µL/min. Peptides were separated with buffer A (1% acetonitrile/0.1% formic acid) and buffer B (80% acetonitrile/0.1% formic acid) with a gradient of 10-60% buffer B over 45 min. Gas and voltage were adjusted as required. MS-TOF scan across (350-1800 m/z) was performed for 0.5 sec for data-dependent acquisition (DDA), followed by DDA MS/MS with an automated selection of top 20 peptides with intensity greater than 100 cps, across 40-1800 *m/z* (0.05 sec per spectrum) using a collision energy of 40 ± 15 V. For data-independent acquisition (DIA) SWATH analyses, MS scans across 350-1800 *m/z* were performed (0.05 sec), followed by high sensitivity DIA mode using 26 *m/z* isolation windows for 0.1 sec, across 400-1250 *m/z*. Collision energy values for SWATH samples were automatically assigned by Analyst software (SCIEX^®^) based on *m/z* mass windows.

### 2.6 Confirmation of differential protein abundance by ELISA

The differential abundance of selected proteins, including alpha-1-acid glycoprotein (AGP), conglutinin (CGN1) and immunoglobulin G (IgG), was confirmed by using commercially available ELISA kits with the serum samples from the relevant groups. The ELISA kits used were Bovine Alpha 1 Acid Glycoprotein ELISA kit (abcam^®^, Cat. No. ab205069, USA), Bovine IgG ELISA kit-45 minutes (abcam^®^, Cat. No. ab273152, USA) and Bovine Conglutinin ELISA kit (MyBioSource^®^, Cat. No. MBS721038, USA) and each assay was performed in duplicate with two replicates, according to the manufacturer’s instructions.

### 2.7 Data analysis

Proteins from DDA data were identified using ProteinPilot software (SCIEX^®^5.02), searching against all bovine proteins in UniProtKB (downloaded 11 May 2021; 46754 total entries), with settings as follows: sample type = identification, cysteine alkylation = iodoacetamide, instrument = TripleTOF5600, species = none, ID focus = biological modifications, digestion enzyme = trypsin, search effort = thorough ID. An ion library from proteins identified with ProteinPilot at 1% false discovery rate was used for analysis of SWATH data. The abundance of peptides in each sample was determined using PeakView 2.1 (SCIEX^®^) as previously described (27), with settings: shared peptides = allowed; peptide confidence threshold = 99%; false discovery rate = 1%; peptides per protein = 6; transitions per peptide = 6; XIC extraction widow = 6 min; XIC width = 75 ppm. The mass spectrometry proteomics data have been deposited to the ProteomeXchange Consortium *via* the PRIDE (28) partner repository with the dataset identifiers PXD036561 and PXD036563 for skin and serum samples, respectively.

A python script (https://github.com/bschulzlab/reformatMS) was used to reformat the PeakView output, with a 1% peptide FDR cut-off to remove the ion measurements for low quality peptides from each sample, and reformatting appropriate for use with MSstats (29). Differences in protein abundance were determined with a linear mixed model using MSstats (2.4) in R (30), with Benjamini and Hochberg corrections adjusting for multiple comparisons and a significance threshold of P < 10^-5^. Proteins and samples were both clustered with Cluster 3.0, applying a hierarchical, uncentered correlation, and complete linkage (31). Search Tool for the Retrieval of Interacting Genes/Proteins (STRING) was used to identify protein-protein interaction and characterization for gene ontology (GO) terms for biological processes (BPs) and Kyoto Encyclopedia Genes and Genomes Pathways (KEGG) using Uniprot accession identifiers of significantly differentially abundant (DA) proteins as a target list (32). The *Bos taurus* genome was used as background in the STRING analysis with the following basic settings: meaning of network edges as evidence; active interaction sources included were experiments, databases, co-expression, neighborhood, gene expression and co-occurrence; high confidence (0.700) for the minimum required interaction score, and *k*-means clustering with the number of clusters set at 3. In addition, for serum samples, over-representation analysis of GO terms was also performed with *clusterProfiler* R package using filtered gene lists (P-value <0.05 and |log_2_FC| >0.3) from pre-exposure vs early (6h PFI) and prolonged exposure (105d PFI) datasets. Graphs were produced with dot plot and category network plot functions from this package. Ingenuity pathway analysis was used to perform pathway enrichment according to biological functions in the Ingenuity Pathways Knowledge Base (Ingenuity Systems, Redwood City, CA). For skin samples, protein class GO terms enrichment was also performed with PANTHER classification system (Gene Ontology Phylogenetic Annotation Project, v 16.0. Available from http://www.pantherdb.org/).

## 3 Results

### 3.1 Tick scoring and resistance ranking

**Figure 1B** shows the mean tick scores for the two groups of cattle over the period of the infestation trial. All the animals carried similar numbers of ticks for the first three scorings, and the scores were less variable between weeks 8 to 15 than in earlier infestations. Therefore, the mean tick score for this period was used to rank the animals. The mean tick score of resistant cattle (1.27 ± 0.29) was significantly lower than that of the susceptible cattle (3.82 ± 0.40) with a P-value < 0.0001 (**Figure 1C**).

### 3.2 Protein identification

ProteinPilot software (SCIEX^®^5.02) search identified a total of 221 and 617 proteins in serum and skin samples, respectively (**Supplementary File 1: Table S1**). SWATH-MS was used to measure the relative abundance of each protein in each individual unpooled sample, quantifying 167 (serum) and 333 (skin) proteins using PeakView 2.1 (SCIEX^®^) at 1% FDR cut-off (**Supplementary File 1; Tables S2, S3**). The serum and skin samples from the two groups of cattle revealed major differences in proteomes before and after exposure to cattle ticks. Statistical comparison of the two groups at different time points provided further insight into the serum and skin proteomes. In the ensuing sections, the following terms are used for simplicity: for significantly differentially abundant proteins, “differentially abundant proteins or DAPs”; for proteins with significantly higher relative abundance, “H-RAPs” and for proteins with significantly lower relative abundance “L-RAPs”.

### 3.3 Variation in serum and skin proteomes

**Figure 2:**
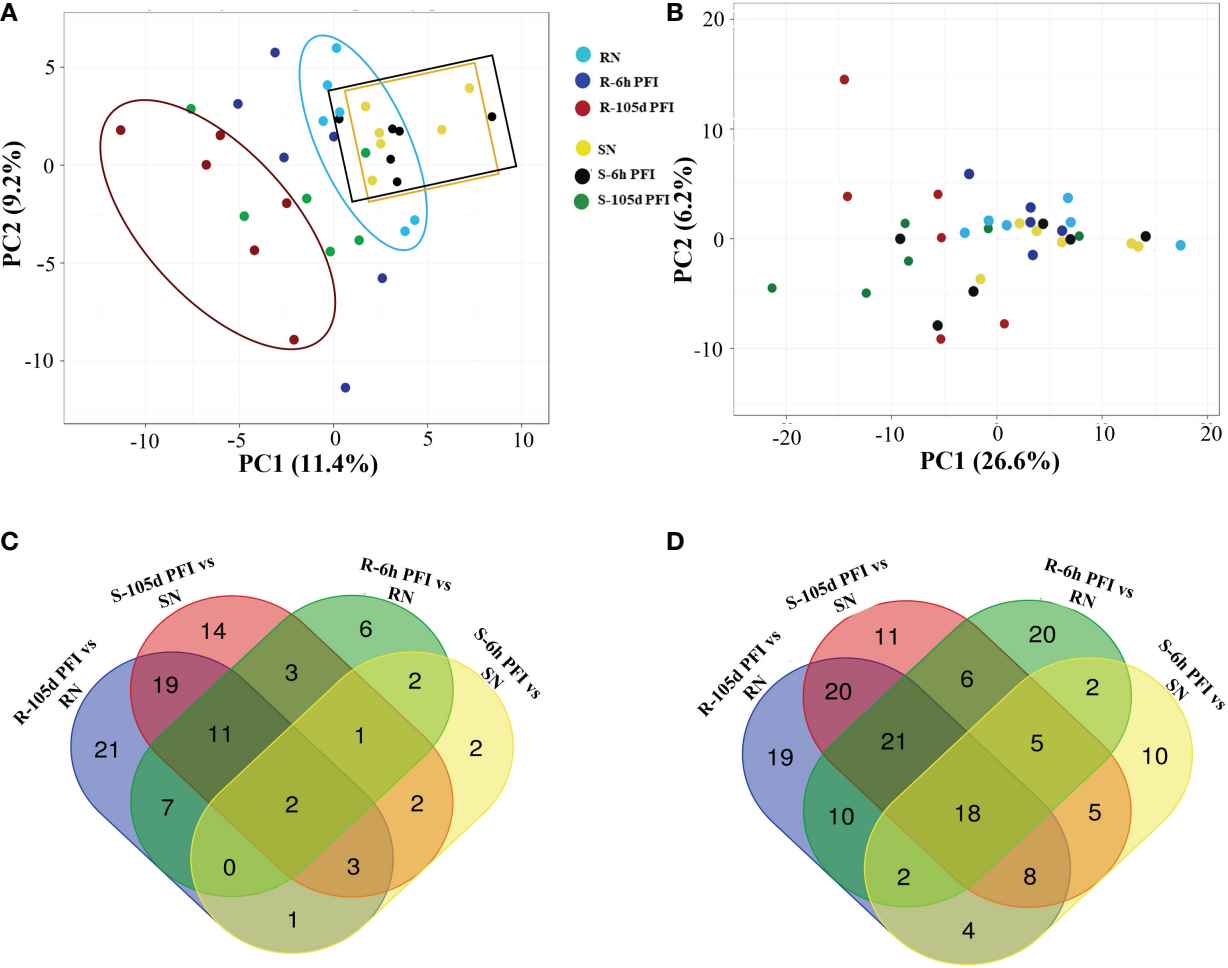
The effect of tick infestation on serum and skin proteomes of resistant and susceptible Brangus cattle. Principal component analysis (PCA) of the log_10_ protein abundances normalised to a total protein abundance in each group for serum **(A)** and skin **(B)** samples; coloured by tick phenotype and the sampling time point: tick resistant naïve (RN) = light sky-blue, tick resistant after 6-hour post-first infestation (R-6h PFI) = blue, tick resistant (R-105d PFI) = red; tick susceptible naïve (SN) = yellow, tick susceptible after 6-hour post-first infestation (S-6h PFI) = black, tick susceptible (S-105d PFI) = green. For PCA with serum samples, the first component (x-axis) accounted for 11.4% of the total variance and the second (y-axis) was 9.2%. For PCA with skin samples, the first component (x-axis) accounted for 26.6% of the total variance and the second (y-axis) was 6.2%. Venn diagrams of the number of proteins significantly (P<10^−5^) different in abundance in serum **(C)** and skin **(D)** samples from different comparisons.

The principal component analysis provided an overview of the variation of peptide abundance between the samples (**Figure 2A, B**). For serum samples from resistant cattle, the PCA showed partial separation and clustering of all three groups, with samples from early exposure (blue dots) clustering between the naïve (light-sky blue) and resistant (red) groups (**Figure 2A**). Samples from susceptible cattle following prolonged exposure (green dots) were separated from susceptible-naïve (yellow) and early exposure (black) samples which were clustered together (**Figure 2A**). The PCA with skin samples showed partial separation and clustering of post-infestation (red and green dots) from naïve and early exposure time point for both resistant and susceptible groups (**Figure 2B**). Many proteins showed significant differences in abundance when samples from tick-exposed cattle were compared with naïve samples from each group, and there were some common proteins between different comparisons in serum and skin samples (**Figure 2C, D**; **Supplementary File 1: Tables S2, S3**).

### 3.4 Effects of tick exposure on host proteome

The changes in serum and skin proteomes following early (6h PFI) and prolonged (105d PFI) exposure of susceptible and resistant cattle to ticks compared to the respective naïve samples represented systemic and local host response to the tick infestation.

#### 3.4.1 Serum samples

**Figure 3:**
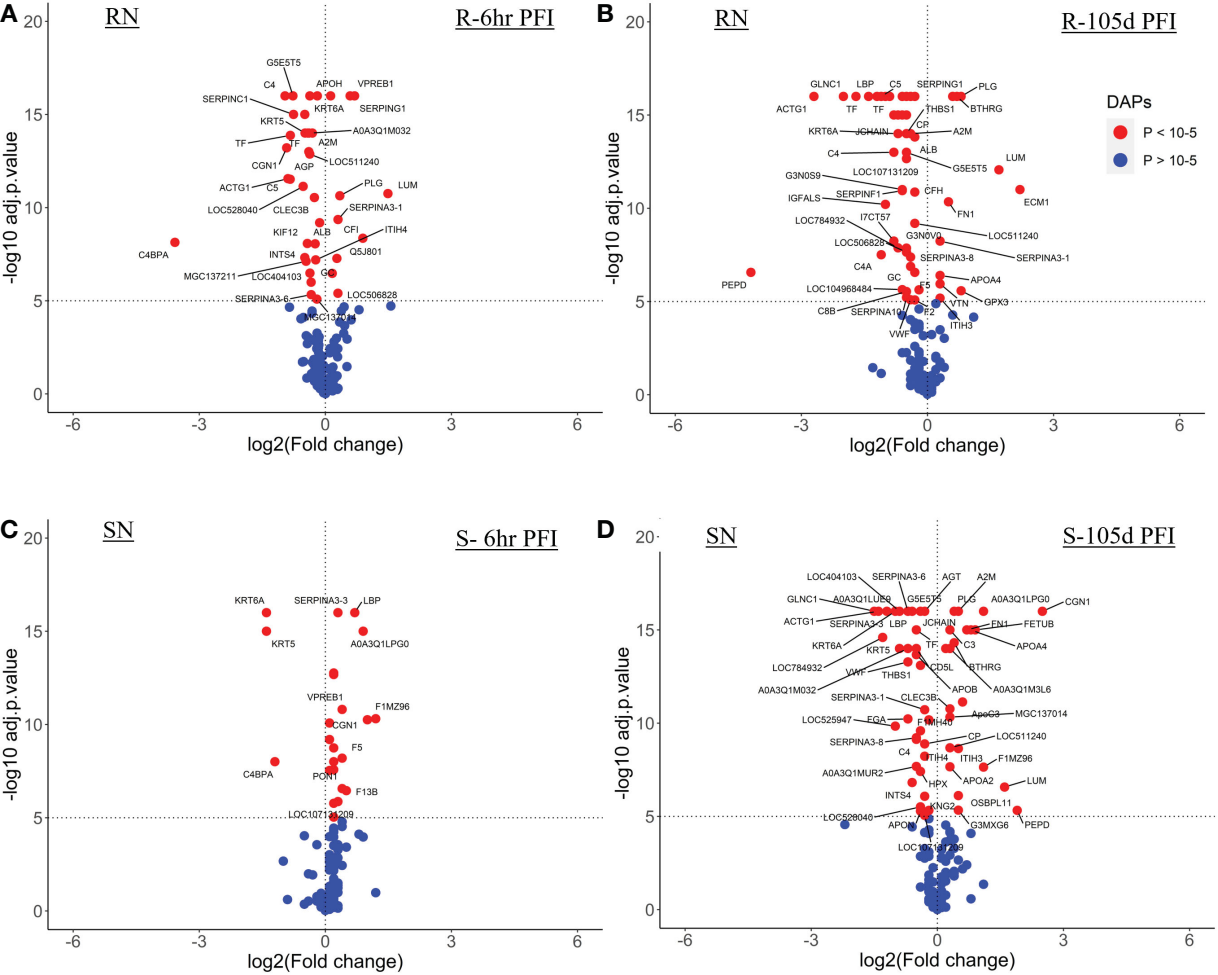
Volcano plots illustrating the differentially abundant proteins in serum samples from tick-resistant and susceptible naïve cattle in response to early (6 hr post-first infestation) and prolonged exposure (105 days post-first infestation) to ticks. DA proteins in: **(A)** resistant naïve compared to early exposure (RN vs R-6h PFI) samples; **(B)** resistant naïve compared to prolonged exposure (RN vs R-105d PFI) samples from resistant cattle; **(C)** susceptible naïve compared to early exposure (SN vs S-6h PFI) samples and **(D)** susceptible naïve compared to prolonged exposure (SN vs S-105d PFI) samples from susceptible cattle. Coloured dots represent proteins significantly different in abundance (P < 10^-5^). Red, significantly different in abundance (P < 10^-5^). Blue, not significantly different in abundance (P > 10^-5^).

The resistant cattle showed higher numbers of DAPs (43 and 62) than susceptible cattle (25 and 58) following early and prolonged tick infestation (**Figure 3**; **Supplementary File 1: Table S4**). Interestingly, the abundance of most of the proteins in resistant cattle was reduced in response to early (31 of 44) and prolonged tick exposure (51 of 62) (**Figure 3A, B**; **Supplementary File 1: Table S4.1, -4.2**). Susceptible cattle showed a slightly different pattern at 6h PFI with 21 of 25 proteins showing higher abundance but 11 proteins with log_2_FC values < 0.3, whereas the abundance of 36 of 58 DAPs was reduced following prolonged tick exposure (**Figure 3C, D**; **Supplementary File 1: Table S4.3, 4.4**).

**Figure 4:**
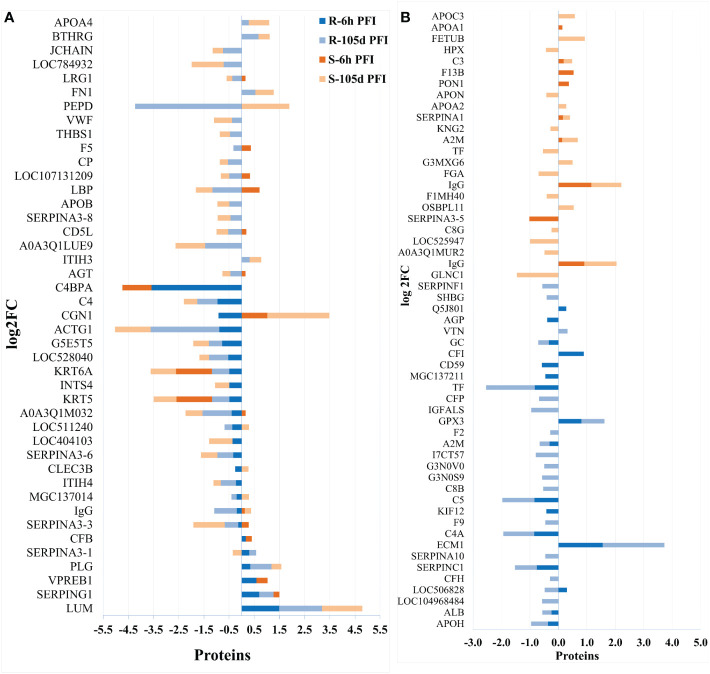
Changes in the relative abundance of proteins commonly **(A)** and uniquely **(B)** manifested in the serum of resistant and susceptible groups relative to their respective naïve groups in response to early (6hr post-first infestation) and prolonged (105d post-first infestation) tick infestation.

**Figure 5:**
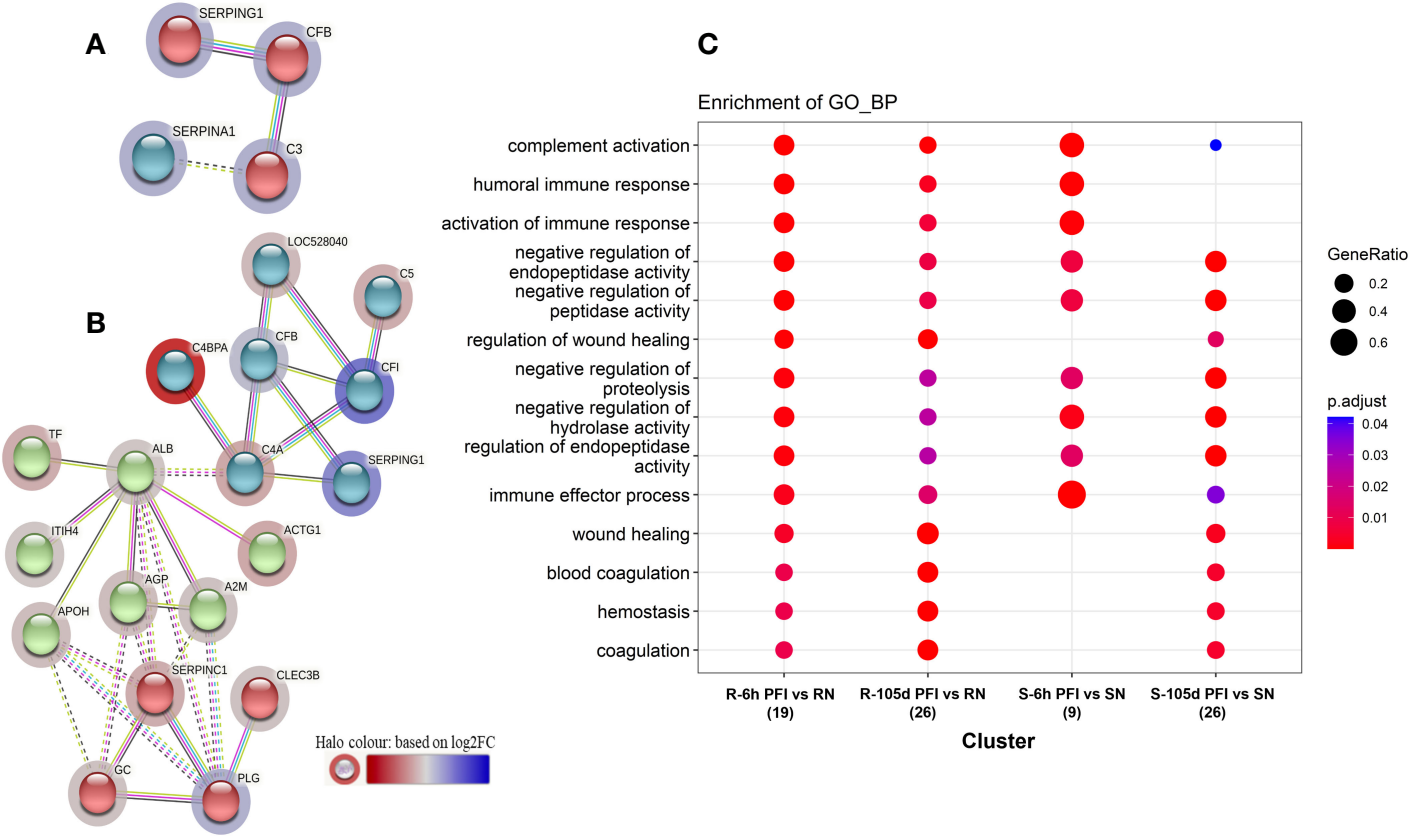
Protein-protein interaction networks among differentially abundant proteins in serum samples of tick-susceptible **(A)** and resistant cattle **(B)** in response to early tick infestation (SN vs S-6h PFI and RN vs R-6h PFI). Each node represents an individual protein. *k*-mean clusters showing strong interactions are highlighted as “red”, “green”, and “cyan blue” coloured nodes. The halo colour is based on the log_2_FC value of the proteins in the dataset. **(C)** Functional enrichment analysis of the differentially abundant proteins in serum samples of tick-resistant and susceptible cattle in response to early (6h PFI) and prolonged (105d PFI) tick infestation. Enriched GO Biological Process terms in the differentially abundant proteins from the comparisons: RN vs R-6h PFI and RN vs R-105d PFI; and SN vs S-6h PFI and SN vs S-105d PFI performed by *clusterProfiler.* The dot colour represents the significance (P-adjusted < 0.05) of the term, and the dot size (GeneRatio) represents the ratio of input genes that are annotated in a term.

Changes in the relative abundance of serum proteins in response to tick infestation in resistant and susceptible groups relative to their respective naïve groups over time are shown in **Figure 4**. For example, in response to early tick exposure (naïve vs 6 hr PFI), the susceptible group showed a higher abundance of immunoglobulin-Gamma (F1MZ96 & A0A3Q1LPG0), conglutinin (CGN1; P23805) coagulation factors F5 (F5; F1N0I3) and F13B (F13B; Q2TBQ1), lipopolysaccharide-binding protein (LBP; F1MNN7), and complement 4-anaphylatoxin (LOC107131209; F1MVK1) (**Figures 4A, B**; **Supplementary File 1: Table S4.1**). Whereas resistant cattle showed a higher abundance of lumican (LUM; Q05443), extracellular matrix protein-1 (ECM1; A5PJT7), complement factor I (CFI; Q32PI4) and plasminogen (P06868) (**Figures 4A, B**; **Supplementary File 1: Table S4.3**). The abundance of pre-B lymphocyte 1 (VPREB1; A0A3Q1LWV4) and Serpin family members (G1 and A3) was higher in both groups. Alternatively, cytoskeletal structural proteins (keratins 5; M0QVZ6 & 6A; M0QVY0) and complement 4 binding protein A (C4BPA; A5D9D2) were the L-RAPs in both groups (**Figure 4B**). In addition, resistant cattle showed a lower abundance of immune response-related proteins, including complement factors (C4, C5a), Gc-globulin (GC; Q3MHN5), CGN1, CD59 glycoprotein (CD59; Q32PA1), blood coagulation proteins including beta-2 glycoprotein-1 (APOH; A0A140T843) and antithrombin-III (SERPINCI; A0A3Q1NJR8), and the acute-phase response protein: alpha-1-acid glycoprotein (AGP; Q5GN72). Functional protein-protein interactions (PPI) for DAPs in susceptible and resistant cattle following early exposure to ticks are shown in **Figures 5A, 5B** representing different *k*-mean clusters in each group. The DAPs in susceptible and resistant cattle were associated with immune system related BP GO terms, including complement activation (GO:0006956) through classical pathway (GO:0006958), leukocyte mediated immunity (GO:0002443) and regulation of immune response (GO:0050776). In addition to these BP GO terms, DAPs in resistant cattle were enriched for blood coagulation (GO:0007596), wound healing (GO:0042060) and inflammatory response (GO:0006954) (**Figure 5C**; **Supplementary File 1: Table S5**). The abundance of most of the proteins contributing to these processes was decreased in response to early tick infestation in resistant cattle. KEGG pathway analysis showed that complement and coagulation cascades (bta04610) were enriched in both groups.

The response to prolonged tick exposure (naïve vs 105 days PFI) showed some similarities in both resistant and susceptible groups, where 29 DAPs were common in the two comparisons (**Figures 4A, B**). For example, the abundance of LUM, PLG, fibronectin (FN1; G5E5B0) and apolipoprotein A-IV (APOA4; V6F7X3) increased in both groups following tick exposure. Whereas 23 proteins showed reduced abundance in both the groups, including actin gamma (ACTG1; P63258), LBP, C4, C4a, CD5 molecule like (CD5; A6QNW7), apolipoprotein B (APOB; E1BNR0) and von Willebrand factor (VWF; F5XVA9) (**Figure 4A**). In addition, the abundance of 29 and 34 proteins showed unique changes in susceptible and resistant groups, respectively (**Figure 4B**). For example, susceptible cattle (compared to susceptible naïve) showed a higher abundance of IgG, CGN1, C3 and apolipoproteins (APOA-II and C-III), and a lower abundance of fibrinogen alpha (FGA; F6QND5) and kininogen 2 (KNG2; P01045) which were not observed in resistant cattle. Similarly, the resistant cattle (compared to resistant naïve) exhibited higher abundance of glutathione peroxidase 3 (GPX3; P37141), vitronectin (VTN; Q3ZBS7) and SERPING1; whereas complement factors (C5a, C8B, CFH, CFP), coagulation factors (F2, F5 and F9), APOH, and pigment epithelium-derived factor (PEDF; Q95121) were the L-RAPs. The PPI analysis generated similar networks for DAPs in susceptible and resistant cattle clustering proteins related to immune responses and blood coagulation into similar clusters (**Supplementary File 2: Figure S1**). The DAPs in both groups contributed to some common BP GO terms, for example, acute phase response (GO:0006953), complement activation (GO:0006956), leukocyte mediate immunity (GO:0002443), blood coagulation (GO:0007596), wound healing (GO:0042060) and inflammatory response (GO:0006954). The abundance of most of the proteins contributing to these processes was decreased in response to prolonged tick infestation in both groups. In addition, the DAPs in susceptible cattle were also enriched in fibrinolysis (GO:0042730) and decreased iron ion transport (GO:0006826) BP GO terms which were not detected in resistant cattle.

#### 3.4.2 Skin samples

**Figure 6:**
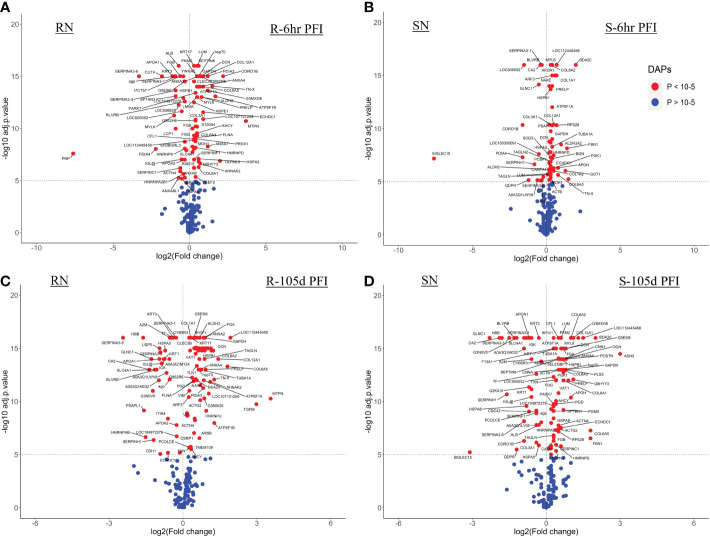
Volcano plots illustrating the differentially abundant proteins in skin samples from tick-resistant and susceptible naïve cattle in response to early (6h post-first infestation) and prolonged (105d PFI) tick infestation. DA proteins in **(A)** resistant naïve compared to early exposure (RN vs R-6h PFI) samples from resistant cattle; **(B)** susceptible naïve compared to early exposure (SN vs S-6h PFI) samples from susceptible cattle; **(C)** resistant naïve compared to prolonged exposure (RN vs R-105d PFI) samples from resistant cattle; and **(D)** susceptible naïve compared to prolonged exposure (SN vs S-105d PFI) samples from susceptible cattle. Coloured dots represent proteins significantly different in abundance (P < 10^-5^). Red, significantly different in abundance (P < 10^-5^). Blue, not significantly different in abundance (P > 10^-5^).

With the skin samples, resistant cattle were more responsive with 94 DAPs (62 H-RAPs and 32 L-RAPs) following early exposure to ticks as compared to 66 DAPs (50 H-RAPs and 16 L-RAPs) in the susceptible group (**Figures 6A, B**; **Supplementary File 1: Tables S6.1, S6.3**). Similarly, a higher number of DAPs were identified in the resistant group (77 H-RAPs and 30 L-RAPs) after prolonged tick exposure (105days PFI) as compared to susceptible cattle (69 H-RAPs and 31 L-RAPs) (**Figures 6C, D**; **Supplementary File 1: Tables S6.2, -6.4**).

**Figure 7:**
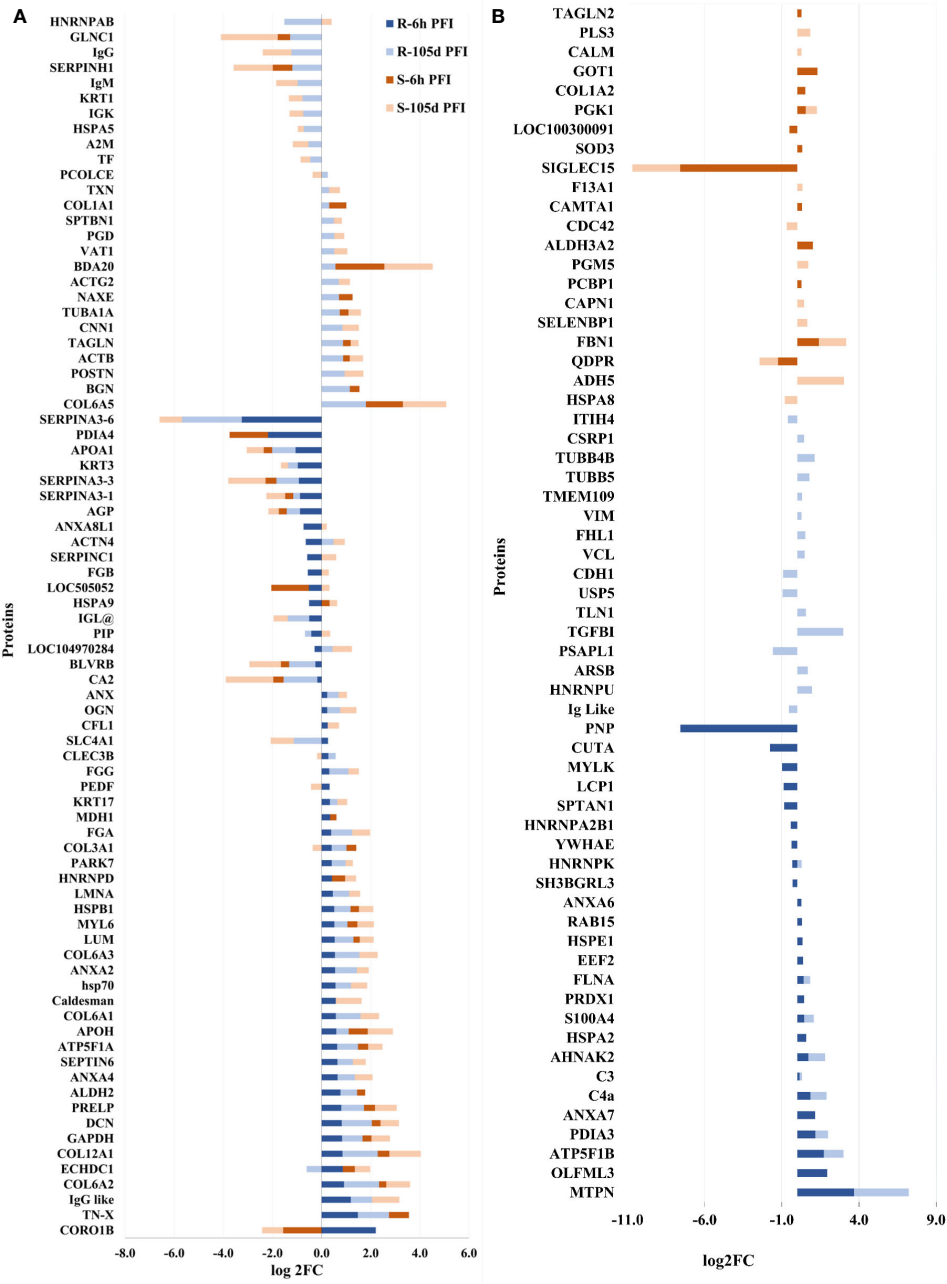
Changes in the relative abundance of proteins commonly **(A)** and uniquely **(B)** manifested in the skin samples of resistant and susceptible groups relative to their respective naïve groups in response to early (6h post-first infestation) and prolonged (105d post-first infestation) tick infestation.

Following early exposure, 26 DAPs were common between the two groups. Different structural proteins, including collagens (COL3A1, COL6A2, COL12A1), LUM, decorin (DCN; P21793) and tenascin X (TN-X; O18977) showed increased levels in both resistant and susceptible cattle with comparatively higher levels (2-3 times) in the resistant group (**Figure 7A**). Coronin 1B (CORO1B; H7BWW0) showed higher abundance (log_2_FC 2.2) in resistant and lower abundance (log_2_FC -1.6) in susceptible cattle, whereas the abundance of stress-70 (HSPA9; Q3ZCH0) was higher in susceptible and lower in resistant cattle at 6 hr PFI. Extracellular matrix proteins comprised 10% of the total proteins identified as H-RAPs in both groups. The abundance of calcium binding proteins (9% of total H-RAPs) also increased in resistant cattle in response to tick infestation. In addition, resistant cattle showed higher abundances of C4, C3, APOH, PEDF (SERPINF1), COL6 (A1 and A3), keratin-17 (KRT17; A0A140T867) and annexin-A family members (A2, A4, A6, A7) proteins following early tick exposure (**Figure 7B**). The functional PPI analysis identified one similar cluster containing proteins contributing to extracellular matrix organization (GO:0030198) (**Supplementary File 2: Figure S2**). This cluster in susceptible cattle also contained proteins involved in collagen fibril organization (GO:0030199) and skin morphogenesis (GO:0043589). The H-RAPs in resistant cattle also contributed to enrichment of immune-related biological processes, such as blood coagulation, fibrin clot formation (GO:0072378), platelet aggregation (GO:0070527) and wound healing (GO:0042060) which were not identified in the susceptible group following early exposure. Pathway analysis identified that platelet activation (bta04611) and ECM-receptor interaction (bta04512) pathways were enriched for proteins in both groups. However, H-RAPs in resistant cattle were also enriched in complement and coagulation cascade (bta04610) and focal adhesion (bta04510) (**Supplementary File 1: Tables S7.1, -7.2**).

The prolonged tick infestation elicited similar skin changes in both groups of cattle with 66 DAPs commonly measured in both resistant and susceptible cattle, and the abundance of most of these proteins increased (**Figure 7A**). Similar to the naïve vs early exposure comparison, the most highly abundant proteins in both groups following prolonged exposure were structural proteins, including collagens (COL6-A1 and COL12-A1), and extracellular matrix proteins, including LUM, prolargin (PRELP; A6QQQ3), annexins (ANX-A2 and A4) and fibrinogens alpha (FGA; F6QND5) and gamma (FGG; Q3SZZ9). In addition, *Bos taurus* major allergen (BDA20; A0A3Q1MH50) was highly abundant only in susceptible cattle after early exposure, while its abundance increased in both susceptible (log_2_FC = 2.0) and resistant (log_2_FC = 0.6) cattle following prolonged exposure to ticks compared to naïve cattle. Some of the common L-RAPs in both groups were carbonic anhydrase 2 (CA2; P00921), SERPIN-A3, collagen-binding protein (SERPIN-H1; Q2KJH6), alpha-2-macroglobulin (A2M; Q7SIH1), APOA1 and AGP. The proteins representing calcium binding class (9% of total H-RAPs) were at higher abundance after prolonged tick infestation in resistant cattle, whereas only two calcium binding proteins were higher in susceptible cattle. In addition, some of the unique proteins in susceptible cattle with higher abundance were antithrombin-III, coagulation factor XIII A chain (F13A1; F1MW44) and fibrinogen beta chain (FGB; A0A3Q1MG04). Similarly, extracellular matrix proteins including biglycan (BGN; P21809) and TN-X, C4, C3, and Vitamin D binding protein (GC; I7CT57) were some of the important unique proteins in resistant cattle, whereas the abundance of APOA2 and two immunoglobulin like proteins was reduced in the skin of resistant cattle in response to prolonged tick infestation (**Figure 7B**).

Functional characterisation and PPI analysis of DAPs at 105d PFI showed that the ECM protein cluster contained a greater number of proteins in resistant cattle than in susceptible cattle (**Supplementary File 2: Figures S3, S4**). In both groups of cattle, H-RAPs contributed to the enrichment of BP processes such as extracellular matrix organization (GO:0030198), blood coagulation, fibrin clot formation (GO:0072378), platelet aggregation (GO:0070527) and wound healing (GO:0042060). In addition, some of the important unique BP GO terms enriched in susceptible cattle were response to stress (GO:0006950), regulation of cellular response to oxidative stress (GO:1900407) and negative regulation of coagulation (GO:0050819). The unique BP GO terms in resistant cattle were cytoskeleton organization (GO:0007010) and collagen biosynthetic process (GO:0032964) (**Supplementary File 1: Tables S7.3, -7.4**). KEGG pathway analysis identified complement and coagulation cascade (bta04610) and ECM-receptor interaction (bta04512) pathways in both groups, whereas platelet activation (bta04611) was only enriched for proteins in resistant cattle.

### 3.5 Comparison of resistant and susceptible cattle proteomes

The serum and skin samples of resistant and susceptible cattle were compared before (naïve) and after (early & prolonged) tick exposure to reveal the differences in proteomic profiles between the two groups.

#### 3.5.1 Comparison of serum samples

**Figure 8:**
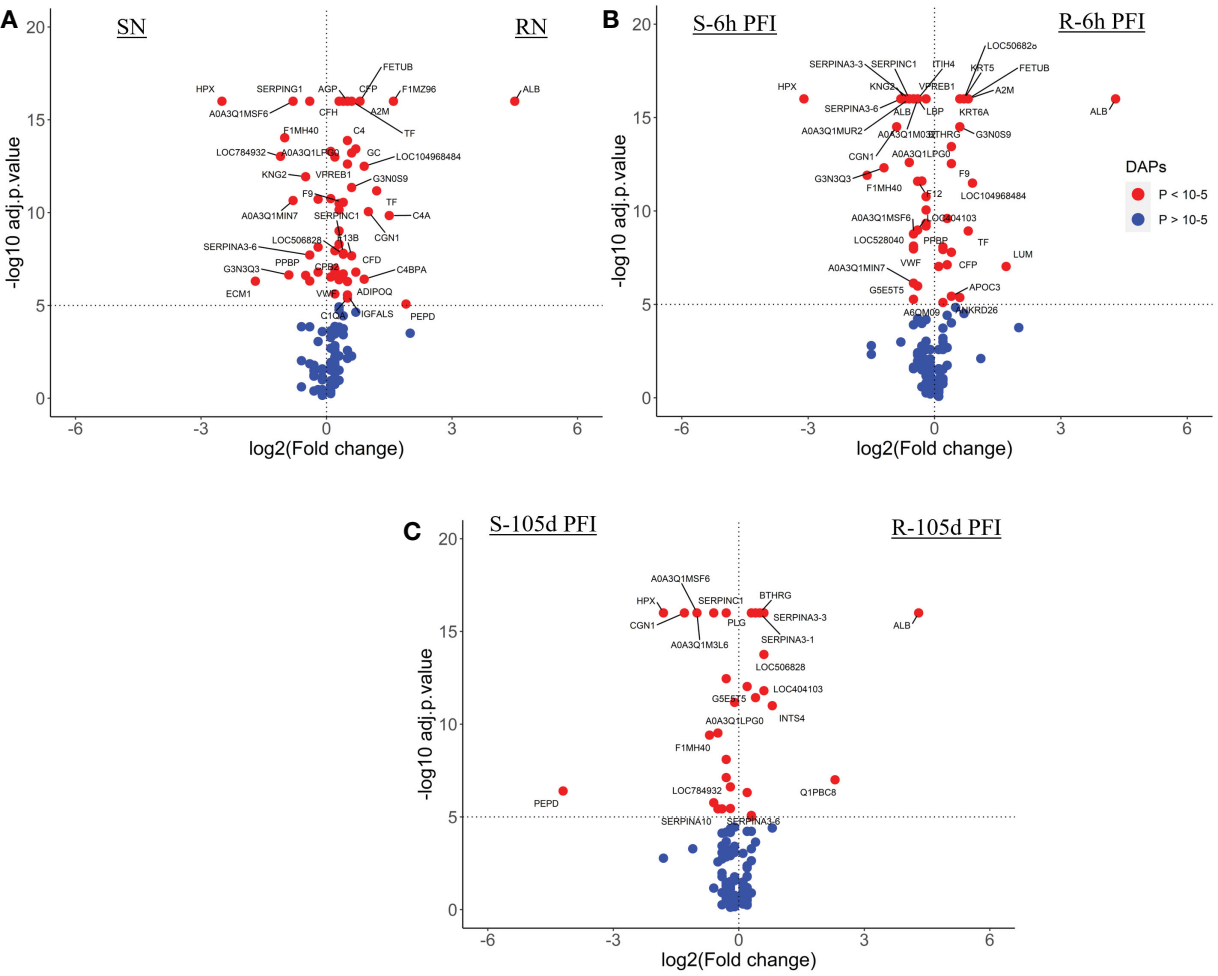
Volcano plots illustrating the DA proteins in serum samples of tick-resistant and -susceptible groups of cattle when compared before and after exposure to cattle ticks. DA proteins in **(A)** susceptible naïve compared to resistant naïve (SN vs RN) samples; susceptible compared to resistant cattle following **(B)** early (S-6h PFI vs R-6h PFI) and **(C)** prolonged S-105d PFI vs R-105d PFI tick infestation. Coloured dots represent proteins significantly different in abundance (P < 10^-5^). Red, significantly different in abundance (P < 10^-5^). Blue, not significantly different in abundance (P > 10^-5^).

**Figure 9:**
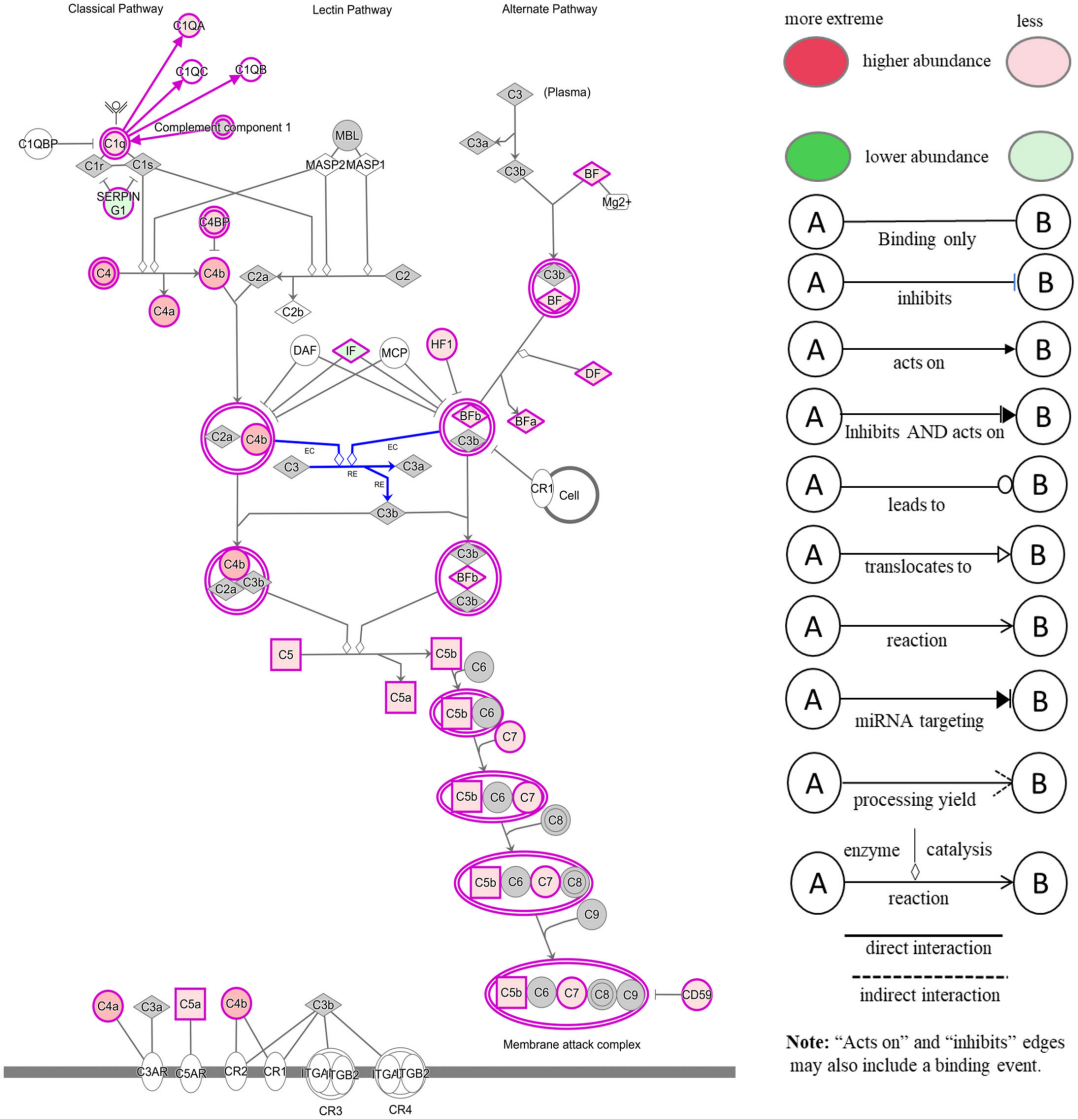
Ingenuity pathway analysis for the significantly differentially abundant proteins in tick-resistant naïve cattle, when compared to susceptible naïve cattle. The DAPs contributed to the complement activation through both classical and alternative pathways. Red boxes indicate proteins with higher abundance and green boxes represent proteins at lower abundance in tick-resistant naïve cattle. White nodes indicate molecules from the knowledge base- not part of the dataset, and the Gray fill color identifies the focus (analysis-Ready) molecules from that dataset.

The comparison of serum samples from both groups of cattle at time 0 (R-naïve vs S- naïve) identified 60 DAPs, with 45 proteins showing higher abundance in resistant cattle (**Figure 8A**). Most of the proteins at higher abundance in resistant naïve cattle were immune response related, for example, complement factors (C1QA, C4, C4a, CFP, CFB, CFH), CGN1, A2M, several immunoglobulin-like proteins, APOA1, APOB and proteins associated with regulation of blood coagulation (F9, F13B and antithrombin III) (**Supplementary File 1: Table S8.1**). Interestingly, resistant naïve animals also showed higher abundances of two uncharacterized proteins, identified as IgG. Susceptible naïve cattle showed higher abundance of fewer proteins that were involved in the regulation of immune response including hemopexin (HPX; Q3SZV7), ECM1, three Ig-like proteins (A0A3Q1MSF6; A0A3Q1MIN7; G3N3Q3), KNG2, C-X-C motif chemokine (PPBP; F1MD83) and VWF. STRING analysis identified distinct clusters of the proteins associated with complement activation and other immune responses, such as regulation of membrane attack complex (MAC) and blood coagulation (**Supplementary File 2: Figure S5A**). Functional characterisation identified 63 BP GO terms, including complement activation (GO:0006956), regulation of membrane attack complex (GO:0001969), regulation of phagocytosis (GO:0050764), innate immune response (GO:0045087), humoral immune response (GO:0006959), leukocyte mediated immunity (GO:0002443) and blood coagulation (GO:0007596). Most of the proteins contributing to these BPs were more abundant in resistant cattle (**Supplementary File 1: Table S9.1**). Complement and coagulation cascade (bta04610) and cholesterol metabolism (bta04979) pathways were enriched in tick-resistant naïve cattle. Of these DAPs, the proteins at higher abundance contributed to complement activation, whereas two proteins at lower abundance were the inhibitory factors of the complement pathway (**Figure 9**).

Following early tick exposure (R-6h PFI vs S-6h PFI), 51 DAPs were identified with 22 H-RAPs in resistant cattle and 29 H-RAPs in susceptible group (**Figure 8B**; **Supplementary File 1: Table S8.2**). Most of the H-RAPs in resistant cattle were the same as observed in resistant naïve cattle, for example, albumin (ALB; B0JYQ0), complement factor P (CFP; Q17QC8), two Ig-like proteins (A0A3Q1MFI7 and A0A3Q1LWV4), APOB, F9, A2M and transferrin (TF; Q29443). Additionally, LUM, KRT5, KRT6A and APOC3 showed higher abundance in resistant cattle as compared to susceptible cattle following early exposure to ticks which were not present in the naïve comparison. Similarly, of 24 H-RAPs in susceptible cattle, 10 proteins, including four Ig-like proteins, KNG-2, PPBP, VWF, SERPIN-A3 and HPX were the same as observed in susceptible naïve samples when compared to resistant naïve. In addition, IgG, five Ig-like proteins, CGN1, antithrombin III, coagulation factor XII (F12; F1MTT3) and acute phase response proteins (APOH and LBP) showed higher abundances in susceptible cattle. Functional analysis showed that resistant cattle carried more H-RAPs contributing to complement activation (GO:0006956), whereas the number of proteins contributing to blood coagulation and humoral immune responses was equal in the two groups. In addition, susceptible cattle showed a greater number of H-RAPs associated with response to stress (GO:0006959), whereas cholesterol homeostasis (GO:0042632) was enriched uniquely in resistant cattle (**Supplementary File 1: Table S9.2**; **Supplementary File 2: Figure S5B**).

The comparison of serum samples at 105d PFI resulted in 33 DAPs between the two groups, with resistant cattle carried 15 H-RAPs as compared to susceptible group (18 H-RAPs) (**Figure 8C**; **Supplementary File 1: Table S8.3**). In resistant cattle, similar to naïve and 6hr PFI comparisons, the abundance of four proteins (ALB, CFB and two Ig-like proteins) was higher, whereas abundance of PLG gradually increased only after tick infestation (6 hr and 105 days PFI). The abundance of monocyte differentiation antigen CD14 (CD14; Q1PBC8), kunitz inhibitor domain-containing protein (BPTI; A0A3Q1M0F4), SERPINs (A3-1 & A3-3) and vitronectin (VTN; Q3ZBS7) was increased in resistant cattle only at 105 days PFI when compared to susceptible cattle. Similarly in susceptible cattle, the abundance of IgG, CGN1, SERPINC1, HPX and three Ig-like proteins was higher in response to early and prolonged tick infestation when compared with resistant cattle. In addition, peptidase D (PEPD; F6Q234) and coagulation factor F9 were highly abundant in susceptible cattle only after prolonged exposure to ticks. Only five BP GO terms were enriched for DAPs in the 105d PFI comparison, blood coagulation (GO:0007596) was active in both groups of cattle, whereas in susceptible cattle a comparatively greater number of proteins contributed to regulation of wound healing (GO:0061041). (**Supplementary File 1: Table S9.3**; **Supplementary File 2: Figure S5C**). Complement and coagulation cascade (bta04610) was the only KEGG pathway enriched in both groups.

#### 3.5.2 Comparison of skin samples

**Figure 10:**
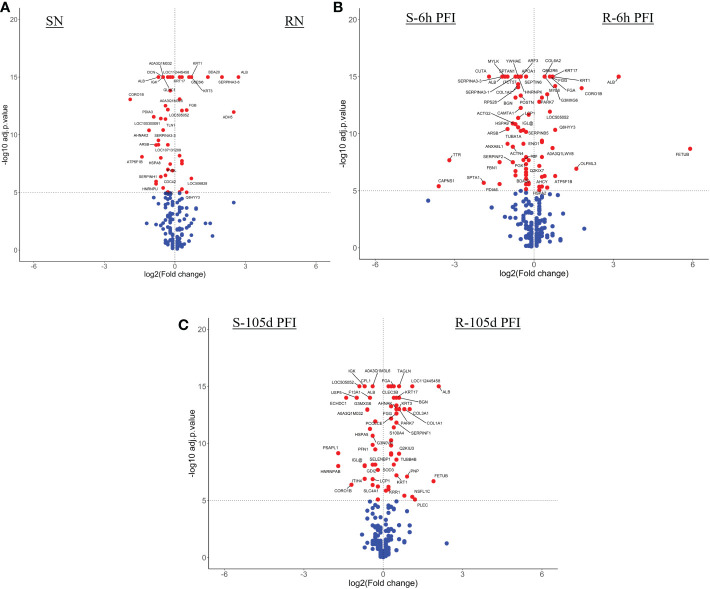
Volcano plots illustrating the DA proteins in skin samples of tick-resistant and -susceptible groups of cattle when compared before and after exposure to cattle ticks. DA proteins in **(A)** susceptible naïve compared to resistant naïve (SN vs RN) samples; susceptible compared to resistant cattle following **(B)** early (S-6h PFI vs R-6h PFI) and **(C)** prolonged S-105d PFI vs R-105d PFI tick infestation. Coloured dots represent proteins significantly different in abundance (P < 10^-5^). Red, significantly different in abundance (P < 10^-5^). Blue, not significantly different in abundance (P > 10^-5^).

The comparison of skin proteome between naïve cattle in both groups measured 63 DAPs with susceptible cattle expressing a relatively greater number of H-RAPs (34) compared to resistant (29) (**Figure 10A**; **Supplementary File 1: Table S10.1**). The major protein classes in both groups were cytoskeletal (19% of total H-RAPs in resistant and 3.0% in susceptible), transfer/carrier (14% of total H-RAPs in resistant and 13% in susceptible), and protease inhibitor proteins (9.5% of total H-RAPs in resistant and 13% in susceptible). For example, the abundances of keratin-1 (KRT1; G3NOV2), keratin-3 (KRT3; G3MXL3), keratin 17 (KRT17; A0A140T867), FGA, FGB and APOH was higher in resistant naïve cattle, whereas susceptible cattle showed higher abundance of CORO1B, C4 isoform, ATP synthase subunit beta (ATP5F1B; A0A452DII8), IgG and F13A1.

Similarly, at 6h PFI comparison, susceptible cattle carried comparatively higher numbers of H-RAPs (51) compared to resistant cattle (35) (**Figure 10B**; **Supplementary File 1: Table S10.2**). There were two protein classes detected as significantly enriched in both groups, with susceptible cattle expressing protease inhibitors (14%) and actin-binding cytoskeletal proteins (12%), whereas resistant cattle expressed calcium-binding proteins (13%) and Hsp70 family chaperone (6.5%) proteins. Some of the proteins with higher abundance in resistant naïve cattle were still higher in resistant cattle at the 6hr PFI comparison with susceptible cattle, for example, KRT1, KRT17 and FGA. In addition, resistant cattle showed higher abundances of C4, fibrinogen gamma (FGG; Q3SZZ9) and FETUB. The susceptible cattle showed higher abundance of acute phase response protein, AGP, IgG, HSPA9, BGN, COL1A2 and alpha-2-antiplasmin (SERPINF2; P28800). In contrast to the first two comparisons, resistant cattle showed a greater number of H-RAPs (44) compared to susceptible cattle (29) at 105d PFI (**Figure 10C**; **Supplementary File 1: Table S10.3**). Based on protein class analysis, extracellular matrix proteins (7.6% of H-RAPs), transfer/carrier proteins (10%) and protease inhibitors (13%) were significantly enriched in resistant cattle, whereas 15% of H-RAPs in susceptible cattle contributed to actin-binding cytoskeletal proteins class. Albumin, keratins (KRT1 and KRT17), COL1A and fibrinogen alpha (FGA) were the highly abundant proteins in resistant cattle at all timepoints (naïve, 6h and 105d PFI) compared to susceptible cattle. The abundance of FETUB, FGG and A2M was significantly higher in resistant cattle after tick infestation at both timepoints (6h and 105 PFI). In addition, ECM proteins including BGN, COL3A, transforming growth factor-beta-induced protein (TGFBI; F1MBS3), PEDF and superoxide dismutase (SOD3) were H-RAPs in resistant cattle only after prolonged tick exposure. Susceptible cattle showed higher abundances of IgG like proteins and F13A1 at all timepoints when compared to resistant cattle. Interestingly, CORO1B and ATP5F1B were higher in susceptible naïve cattle, whereas their abundance increased in resistant cattle following tick infestation at both time points.

Protein-protein networking analysis grouped the proteins associated with blood coagulation, metabolic processes, and structural proteins into similar clusters in both groups of cattle (**Supplementary File 2: Figures S6A–C**). Functional analysis showed that resistant cattle expressed higher numbers of proteins associated with blood coagulation, fibrin clot formation (GO:0072378) and platelet activation (GO:0030168) at all time points as compared to susceptible cattle. In addition, H-RAPs in resistant naïve cattle and 6hr PFI were enriched in complement activation (GO:0006956). Resistant naïve cattle expressed greater numbers of proteins involved in wound healing (GO:0042060), where this BP GO term was equally enriched in both groups at 6h PFI time points. On the other hand, highly abundant proteins in susceptible cattle at 6hr PFI contributed to extracellular matrix organization (GO:0030198) and acute-phase response (GO:0006953) BP GO terms. KEGG pathway analysis showed that complement and coagulation cascades (bta04610) were enriched in resistant cattle following tick infestation at both time points (**Supplementary File 2: Table S11**).

### 3.6 Confirmation of differential protein abundance by ELISA

**Figure 11:**
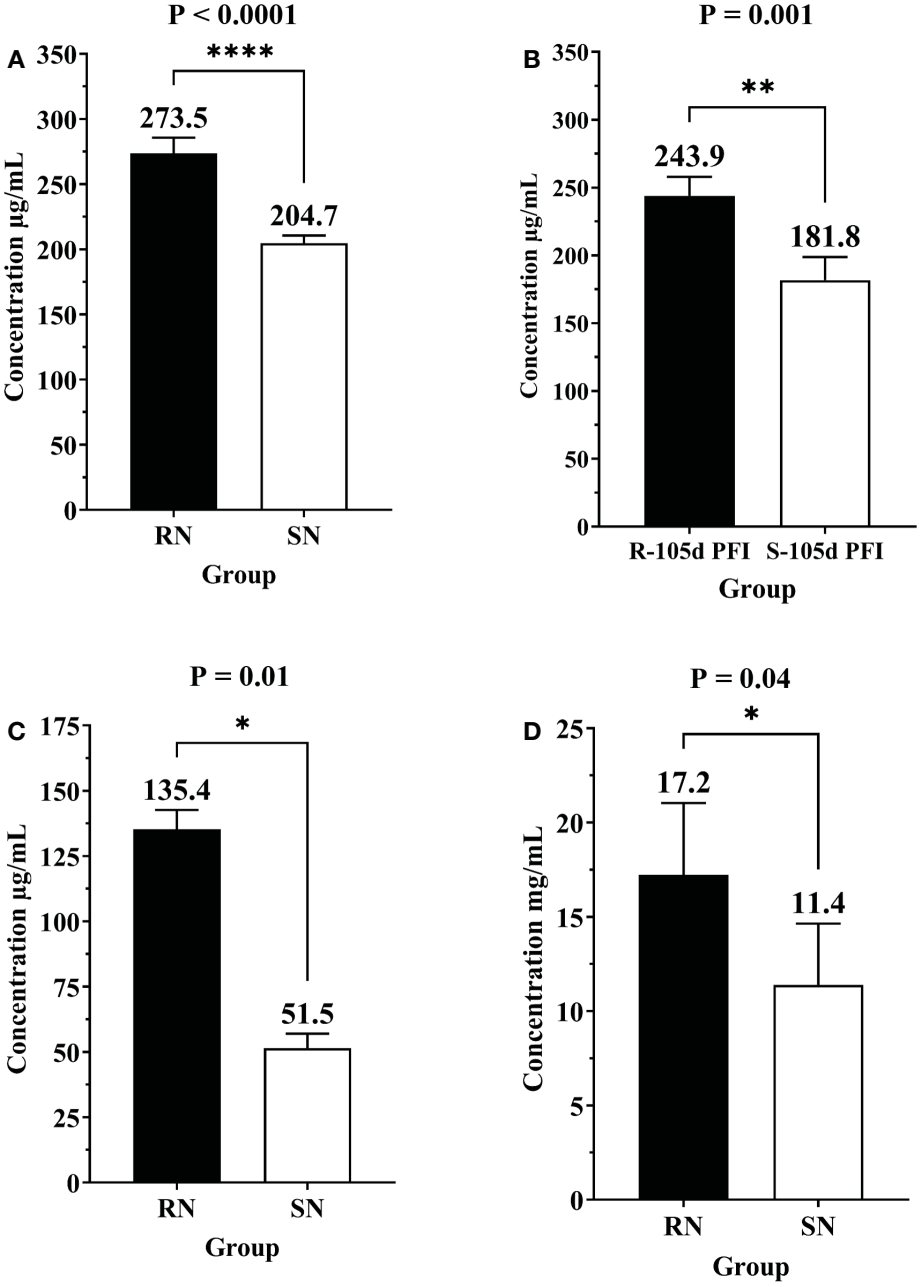
Bar graphs showing the concentrations of alpha-1-acid glycoprotein (AGP), conglutinin (CGN1) and immunoglobulin G (IgG) in sera of different groups of cattle measured by commercially available ELISA kits. Concentration of of alpha-1-acid glycoprotein (AGP) in **(A)** resistant naïve compared to susceptible naïve (RN vs SN), and **(B)** resistant vs susceptible cattle at 105d PFI (R-105d PFI vs S-105d PFI). Concentration of CGN1 **(C)** and IgG **(D)** in resistant naïve compared to susceptible naïve cattle (RN vs SN). Black bars represent resistant cattle; empty bars represent the susceptible group. Asterisk * indicate the level of significance with * = P ≤ 0.05, ** = P ≤ 0.001, and **** = P ≤ 0.0001.

The differential abundance of CGN1, AGP and IgG as determined by ELISA in serum samples from different comparisons confirmed the pattern of relative abundance measured by DIA (**Table 1**). For example, the levels of AGP were significantly higher in resistant naïve (1.3-fold; P < 0.0001) compared to susceptible naïve group as well as resistant 105d PFI (1.3-fold; P = 0.001) compared to susceptible cattle at 105d PFI (**Figures 11A, B**). In addition, CGN1 (2.6-fold; P = 0.010) and IgG (1.5-fold; P = 0.044) were also significantly higher in resistant naïve compared to susceptible naïve cattle (**Figures 11C, D**), respectively. However, the concentration of CGN1 was not significantly different between resistant and susceptible groups at 6hr and 105d PFI timepoints, which contrasted with the MS findings. When compared to the baseline samples, concentrations of AGP (1.4-fold; P = 0.004) and CGN1(2.0-fold; P = 0.02) were significantly lower in resistant cattle following early exposure (**Supplementary File 2: Figure S7A**). In contrast, the concentrations of CGN1 and IgG in susceptible cattle increased in response to early (CGN1 = 1.4-fold with P = 0.004; IgG = 1.5-fold with P = 0.07) and prolonged (CGN1 = 1.6-fold with P = 0.006; IgG = 1.5-fold with P = 0.036) tick infestation (**Supplementary File 2: Figure S7B**). All these findings confirmed the pattern of abundance of these proteins and validated the findings of DIA.

**Table 1:** ELISA confirmation of the changes in the relative abundance of different proteins measured in mass spectrometry.

Protein	Comparison	Relative abundance in MS (Log_2_FC)	Results of ELISA		Agreement
			Fold change	P value	
AGP	RN vs SN	0.5 in RN	1.3 in RN	<0.0001	Yes
	R vs S	0.2 in R	1.3 in R	0.001	Yes
	RN vs R6	0.4 in R6	1.4 in R6	0.004	Yes
CGN1	RN vs SN	1.0 in RN	2.6 in RN	0.01	Yes
	R6 vs S6	0.9 in S6	1.1 in S6	0.104	Yes*
	R vs S	1.3 in S	No difference		No
	RN vs R6	0.9 in R6	2.0 in R6	0.02	Yes
	SN vs S6	1.0 in S6	1.4 in S6	0.004	Yes
	SN vs S	2.5 in S	1.6 in S	0.006	Yes
IgG	RN vs SN	1.6 in RN	1.5 in RN	0.04	Yes
	SN vs S6	1.2 in S6	1.5 in S6	0.07	Yes
	SN vs S	1.1 in S	1.5 in S	0.036	Yes

RN, resistant naïve; R6, resistant 6hr PFI; R, resistant 105d PFI; SN, susceptible naïve; S6, susceptible 6hr PFI; S, susceptible 105d PFI.

* Statistically non-significant.

## 4 Discussion

Given the rapid spread of acaricidal drug resistance, the societal preference for chemical-free animal products, and the lack of an immunogenic anti-tick vaccine, selection for host resistance to ticks offers a sustainable alternative to tick control and can complement existing strategies. The contribution of non-immune factors, including skin thickness and grooming behaviour as well as adaptive immune components (humoral and cellular response) to tick resistance in cattle has been studied extensively (11, 13, 33, 34). However, knowledge about the local and systemic protective responses in a broader context is limited. This large-scale proteomics study reported the variation of serum and skin proteomes in Brangus (*Bos taurus* x *Bos indicus*) cattle to identify the differences in responses of tick-resistant and -susceptible cattle to tick infestation (compared to naïve baseline samples), and the relative abundances of proteins between the two groups before and after tick infestation, thus identifying proteins that could be used as potential biomarker(s) for tick resistance. The PCA showed substantial changes in the proteomes of serum samples of resistant cattle following early and prolonged tick infestation, however, such variations were only observed in susceptible cattle after prolonged tick infestation. This suggests that resistant cattle responded faster to tick infestation as compared to susceptible cattle and these changes in proteome profiles of resistant cattle after prolonged infestation further discriminated them from naïve cattle. This suggests that tick-resistant and susceptible cattle responded differently to tick infestation and these differences in proteome response might be useful to discriminate between susceptible and resistant cattle. The changes in the proteomes in sera occur very early during tick infestation compared to the skin samples, and as the blood is easier to collect, serum samples may offer an advantage for investigating biomarker(s) associated with tick resistance.

### 4.1 Immune response related proteins

The findings showed that following early exposure to ticks, DAPs in animals of high and low resistance were associated with immune response-related GO terms, for example, complement activation and regulation of immune responses. Susceptible cattle showed increased abundances of immune-related proteins, and the log2FC values for most of the proteins were < 0.3, whereas the abundances of immune response-related proteins including complement factors (C4, C4a, C5) and A2M were reduced in resistant cattle. Interestingly, resistant cattle also showed reduced abundances of immune response inhibitory proteins, for example, CD59 glycoprotein (MAC inhibitory factor) and C4BPA, suggesting that resistant cattle developed a balanced immune response at the early stages of tick infestation. Similarly, the reduced abundance of immune response-related proteins was observed in response to prolonged tick infestation in both groups of cattle. These results are in partial agreement with our previous findings in Santa Gertrudis cattle, where some immune-related proteins were reduced in response to prolonged tick exposure in both resistant and susceptible cattle, but, the levels of five complement factors were increased (16). Recently, Mantilla Valdivieso et al. (25) reported that the leukocyte genes involved in immune responses including complement and coagulation cascades and cell signalling pathways were downregulated in both high and low tick-resistant Brangus cattle over short (3 weeks) and long (12 weeks) infestation periods. The authors suggested that migration of the cell populations expressing these genes out of the peripheral circulation may have caused the pathway downregulation. In contrast, proteomic profiling of plasma exosomes showed that highly tick-resistant *Bos taurus* cattle carried proteins associated with immunity and this class was not detected in low-tick-resistance cattle (35). However, that study relied on post-exposure samples and did not analyse or compare the samples collected from tick naïve cattle. Secondly, the study included only three cattle in each group, which might not have captured the true biological variations between the two groups with high confidence.

It is believed that ticks release immunomodulatory compounds in saliva to evade the host immune response and to enable blood-feeding. For example, disruption of complement cascade components, complement pathway enzymes (C2, factors B, C and D) and cell signalling molecules has been reported (reviewed by 36). Silva et al. (37) found that the saliva of *R. microplus*, a very closely related tick was able to inhibit the classical and alternative pathways of the complement system *in vitro*. In the present study, a comparison of naïve serum samples from resistant and susceptible cattle identified an increased abundance of immune-related proteins (including eight complement factors) in resistant naïve cattle. These proteins contributed to complement activation, regulation of activation of membrane attack complex, innate immune response, regulation of phagocytosis and leukocyte-mediated immunity. These findings confirmed the transcriptomics results reported recently (25), where the differences in leukocyte gene expression between the low and high tick resistance cattle were the highest in naïve cattle. Significantly higher abundance of complement factor proteins in resistant cattle is supported by previous research demonstrating the increased expression of genes encoding multiple complement factors in crossbred resistant cattle (38, 39). Furthermore, the analysis of serum samples at other time points (6hr and 105d PFI) showed that susceptible cattle developed these immune responses slowly over time. For example, susceptible cattle showed higher abundances of several immunoglobulin-like proteins including C3 isoform following early and prolonged tick infestation. Increased expression of humoral immunity genes has previously been reported in low resistance cattle (25, 39), but this type of humoral response has not been associated with protection against ticks, and it was suggested as a part of the immunopathology in susceptible hosts (13).

The relatively higher abundance of seven immune response-related proteins in serum samples of susceptible cattle following early exposure suggests that the reduced abundance of immune-related proteins, especially complement factors, in resistant cattle following tick exposure may not be due to the immunomodulatory effects of tick saliva. This might be explained in two ways; first, the relatively lower abundances of complement factors could be associated with complement consumption through non-specific stimulation by parasite enzymes and subsequent effects of kinin and thrombin activation (40, 41). Secondly, this could also be due to the transmigration of these proteins to the skin to elicit an anti-tick response at the tick feeding sites. This hypothesis was confirmed by the detection of higher abundances of C3, C4 and other complement activation proteins including IgM and APOH in the skin of resistant cattle following early and prolonged exposure to ticks which were absent in susceptible cattle. It should also be noted that these proteins were detected in the skin samples which are enriched with highly abundant structural proteins including keratins and collagens, thus, there might be other immune related proteins which might have been obscured by these highly abundant proteins. The deposition of complement components in the epidermal vesicles beneath *Dermacentor andersoni* larval bites has been reported in tick-resistant hosts (42). Given that complement system acts as the first line of host defense to eliminate the invading parasites by forming the membrane attack complex (reviewed by 43), the increased levels of complement factors in the presence of complement activation proteins and reduced levels of CD59 (MAC inhibitor) in resistant cattle following early infestation may result in efficient larval rejection. This argument is supported by previous studies which reported that the highest larval rejection occurs within first 24 hrs following tick application (5, 44). Our data shows that there is a high level of innate immune response in tick-resistant animals and these findings are consistent with the literature which shows that complement plays an important role in tick rejection in many species, including cattle (38, 45, 46).

### 4.2 Proteins associated with hemostasis and wound healing

Tick saliva comprises a cocktail of pharmacologically active compounds which are excreted at the bite site to deploy several proteolytic pathways that modulate the host hemostatic response against tick feeding (47). This results in delayed wound healing and impaired blood clotting which facilitates tick feeding. The host hemostatic mechanisms are indirectly inhibited by the protease inhibitors in tick saliva through blocking the active sites, exosites and receptors of regulatory factors involved in hemostasis, for example, thrombin, factors V, Xa, kallikrein and kallikrein-associated factors (48). Therefore, active blood coagulation processes in the host would hinder the supply of a blood meal to ticks. Our results showed that tick-resistant naïve cattle expressed a greater number of proteins at significantly higher abundances contributing to blood coagulation when compared to susceptible naïve cattle in both serum and skin samples. For example, the abundance of carboxypeptidase B2, also known as thrombin-activatable fibrinolysis inhibitor was higher in resistant naïve cattle. The abundance of carboxypeptidase B2 was also higher in resistant cattle following early and prolonged tick exposure but the differences were not statistically significant (P > 10^-5^). A carboxypeptidase inhibitor protein family identified from *Rhipicephalus bursa* ticks has shown the ability to stimulate fibrinolysis *in vitro* (49). The authors suggested that tick carboxypeptidase inhibitor may contribute to the maintenance of blood flow during feeding and inside the host by the stimulation of fibrinolysis.

Following early and prolonged tick infestation, DAPs in resistant cattle (compared to resistant naïve) were significantly enriched in blood coagulation, hemostasis and wound healing BP GO terms, whereas these processes were enriched in susceptible cattle only after prolonged tick exposure (compared to susceptible naïve). The reduced abundance of three coagulation factors (F2, F5 and F9) in resistant cattle at 105d PFI might be due to the reduced tick challenge, as the last infestation was administered four weeks before this sampling timepoint and progressively low numbers of ticks developed on the skin of resistant cattle compared to susceptible cattle. However, the abundance of antithrombin-III, a negative regulator of blood coagulation was also reduced in resistant cattle compared to pre-infestation and susceptible cattle. The high levels of APOH, one of the three proteins that can up- and down-regulate the complement and coagulation systems were detected in the skin samples of both groups (50). These findings are consistent with previous studies that reported higher expression of genes contributing to blood coagulation and wound healing in tick-resistant cattle (23, 38). Fibrinogens (A, B and G), plasma proteins that act as a bridge between the activated platelets and thus play a major role in blood coagulation, platelet aggregation and vasoconstriction (51), were significantly higher in the skin of resistant naïve (compared to susceptible naïve) as well as in resistant cattle following early and prolonged tick exposure (compared to resistant naïve). Similarly, the highly abundant proteins in the skin of resistant cattle following early and prolonged exposure to ticks contributing to platelet aggregation might have prevented tick feeding from the very first tick infestations. On the other hand, in susceptible cattle, high levels of these proteins were detected only after prolonged exposure. Tick salivary SERPINs from *R. microplus* (RmS-1, RmS-3, RmS-6 and RmS-17) have been shown to reduce platelet aggregation, suggesting a potential role of these proteins in successful feeding activity (52, 53). Platelets play an important role in mammalian hemostasis as platelet plugs are formed as early as four seconds after vascular injury, followed by deposition of fibrin meshwork around the platelet plug to stabilize and hold the platelet plug to the injury site (51). Hence, the intrinsic or induced greater abundance of proteins involved in the hemostasis triad i.e., blood coagulation, platelet aggregation and vasoconstriction in tick-resistant cattle seem to be a counter mechanism for tick feeding strategies.

### 4.3 Calcium binding proteins

Another finding of this study was the significant enrichment of calcium (Ca^2+^) binding proteins (9% of H-RAPs) in the skin samples of tick-resistant cattle in response to early exposure to ticks, whereas this protein family was not detected as differentially abundant in susceptible cattle when compared to their relevant baselines. Similarly, Ca^2+^ binding proteins were also higher in resistant cattle when compared to susceptible cattle at 6h PFI; however, at 105d PFI comparison, resistant cattle carried fewer DAPs representing this class. The primary function of Ca^2+^ binding proteins is to bind Ca^2+^ either for storage or to participate in Ca^+^ signalling pathways. There is a discrepancy between the association of Ca^2+^ signalling, Ca^2+^binding proteins and/or Ca^2+^ ion channel genes with tick resistance phenotypes published previously. For example, increased expression of Ca^2+^ signalling genes was reported in Belmont red and Brahman cattle resistant to ticks in Australia (9, 54). In contrast, higher expression of genes related to Ca^2+^ ion control has been reported in susceptible cattle in Brazil however these studies were not undertaken with tick naïve samples (38, 55). Therefore, the role of calcium signalling, calcium binding, and/or calcium ion control genes in tick resistance needs further investigation using tick naïve cattle and different breeds.

### 4.4 Extracellular matrix and structural proteins

Extracellular matrix (ECM) and structural proteins showed differences in abundance in the skin of both tick-resistant and susceptible cattle in response to tick infestation. However, resistant cattle showed a greater number of ECM proteins at higher abundance following tick infestation. For example, resistant cattle showed significantly higher abundances of multiple members of collagen and annexin-A protein families which were not significantly DA in susceptible cattle at early infestation. Annexin-A plays a role in formation of cell membrane and cytoskeleton and contributes to stabilization of lipid bilayer (reviewed by 56). Members of annexin-A protein family also contribute to the regulation of the integrity of the ECM, and the regulation of cell signal transduction and inflammation (reviewed by 57). For example, ANX-A2 regulates vascular integrity by interacting with actin and adherens junction vascular endothelial cadherin. In addition, ANX-A2 also contributes to the initiation of angiogenesis that promotes tissue repair (58). Previously, Moré et al. (24) reported an upregulation of ANX-A8 gene in the skin of Braford resistant cattle compared to susceptible cattle before and after tick exposure. Although, annexin-A is important in maintaining cellular integrity, the role of annexin-A in tick resistance is not clear. In addition, ECM organization and ECM receptor signalling pathway were enriched for H-RAPs in resistant cattle in response to early and prolonged infestation, whereas these were observed in susceptible cattle only after prolonged tick infestation. Several studies have reported upregulated genes encoding constituents of ECM including keratins and collagens in tick-resistant cattle breeds post-tick exposure (6, 9, 12). It is worth noting that all these studies compared *Bos taurus* with *B. indicus* cattle and did not compare resistance levels within the same breeds.

The abundance of ECM proteins including KRT1, KRT3, KRT17 and COL1A1 was also higher in resistant-naïve samples when compared to susceptible naïve group. Keratins are regulated by activated keratinocytes and contribute to intracellular signalling pathways, for example, protection from stress, and wound healing (reviewed by 59). Epidermal keratin encoding genes (KRT5 and KRT14) have been previously reported as upregulated in tick-resistant Belmont red cattle (10). The authors suggested that epidermal permeability barrier may play an essential role in conferring greater resistance to tick infestation in cattle. In contrast, keratin transcripts were reduced in both low- and high-resistance cattle in response to tick infestation (39). Keratin-1 is present in suprabasal level of the epidermis and plays a major role in epidermal barrier formation to maintain skin integrity and control inflammation (60). In addition, KRT17 is upregulated in response to epidermal barrier breach along with KRT6/16 and stimulates proliferation, cell adhesion, migration, and inflammatory features (reviewed by 59). It is interesting to note that KRT17 abundance was significantly higher in resistant cattle at all timepoints when compared to susceptible cattle, as well as following early and prolonged exposure to ticks compared to the naive expression levels in resistant cattle. It has been reported that the higher expression of KRT6, 16 and 17 is maintained throughout the epidermal remodelling phases until the barrier junction is revived, indicating the importance of these proteins in wound healing (61, 62). Similarly, collagens play a vital role in wound healing through the stimulation of platelet aggregation and thus are important in skin integrity. A tick adhesion inhibitor in the soft tick *Ornithodoros moubata*, has been shown to impair platelet aggregation by inhibiting the platelet adhesion to collagen through interacting with specific integrins (63).

In addition to the structural proteins responsible for skin integrity, the abundance of some other ECM proteins contributing to wound healing was also higher in the skin of resistant cattle. For example, levels of transforming growth factor-beta-induced protein (TGFBIp) and pigment epithelium-derived factor (PEDF) were increased over baseline levels in resistant cattle in response to tick infestation and were higher when compared with susceptible cattle after tick exposure. The transforming growth factor-beta-induced protein is an ECM protein associated with adhesion, migration, proliferation and differentiation of various cells (reviewed by 64). In humans, TGFBIp induced by TGF-β is present in platelets and has been reported to play an essential role in platelet activation by binding on the platelet surface, thus promoting thrombogenesis and wound healing (65). Similarly, PEDF, a multifunctional protein with anti-angiogenic activity, also plays an essential role in skin wound healing by increasing cellular adhesion to regulate keratinocyte migration (66). Decreased PEDF expression was observed in human keratinocytes due to mechanical injury *in vitro*, and the PEDF level was increased in the tissue remodelling phase. It has been previously reported that disintegrin- or thrombospondin-like molecules produced by hard ticks including *Rhipicephalus* spp. can bind to growth factors including TGFB1 and PEDF, thus impeding ECM interactions and angiogenesis which ultimately affects wound healing activities of the host (67, 68). Therefore, highly abundant ECM proteins contributing to skin/epidermal integrity, platelet activation and aggregation as well as wound healing in tick-resistant cattle are potential contributors to tick resistance.

## Conclusions

This study is the first to report the variation in abundance of serum and skin proteins in proteomic profiles of tick-resistant and susceptible cattle in response to tick infestation at a comprehensive level. It also documents differences in serum and skin proteomes between resistant and susceptible cattle prior to and post-tick infestation. The findings show that host responses to tick infestation at systemic and cutaneous levels are detectable as early as six hours after infestation. The skin proteomics data showed that the resistant cattle effectively mobilized the potent immune-related proteins to the skin, at the tick-host interaction site, which along with ECM proteins associated with the maintenance of skin integrity and wound healing increased the likelihood of early larval rejection. The study concludes that the higher abundance of proteins associated with innate immune response, hemostasis, and wound healing at both systemic and cutaneous levels in resistant cattle enables them to develop a strong protective response to tick infestation. Therefore, immune response related proteins such as C4, C4a, AGP and CGN1 (naïve samples), CD14, GC and AGP (post-infestation), should be further explored as potential biomarkers for tick resistance. Most importantly, the differences in the abundance of some proteins were confirmed using ELISA which further supports the potential application of these biomarkers to predict tick resistance.

## Data availability statement

The datasets presented in this study can be found in online repositories. The names of the repository/repositories and accession number(s) are PXD036561 and PXD036563 (PRIDE partner repository).

## Ethics statement

The animal study was reviewed and approved by The University of Queensland Animal Ethics committee.

## Author contributions

AR designed and executed proteomics experiment, data analyses and manuscript writing. AN conducted the mass spectrometry analysis. BS supervised the SWATH analyses. EK helped in data visualization. CC, NJ and AT reviewed the manuscript. AR and AT designed the tick infestation trial undertaken by AR, MK, MN and EM. AT and PJ conceived the research grant. All authors contributed to the article and approved the submitted version.
