# Supplementary material for: Application of quantitative proteomics to discover biomarkers for tick resistance in cattle
Source: Front Immunol. 2023 Jan 30;14:1091066. doi: 10.3389/fimmu.2023.1091066 (PMC9924087; doi:10.3389/fimmu.2023.1091066)
Supplement: Supplementary file 2 [file DataSheet_2.docx]

**
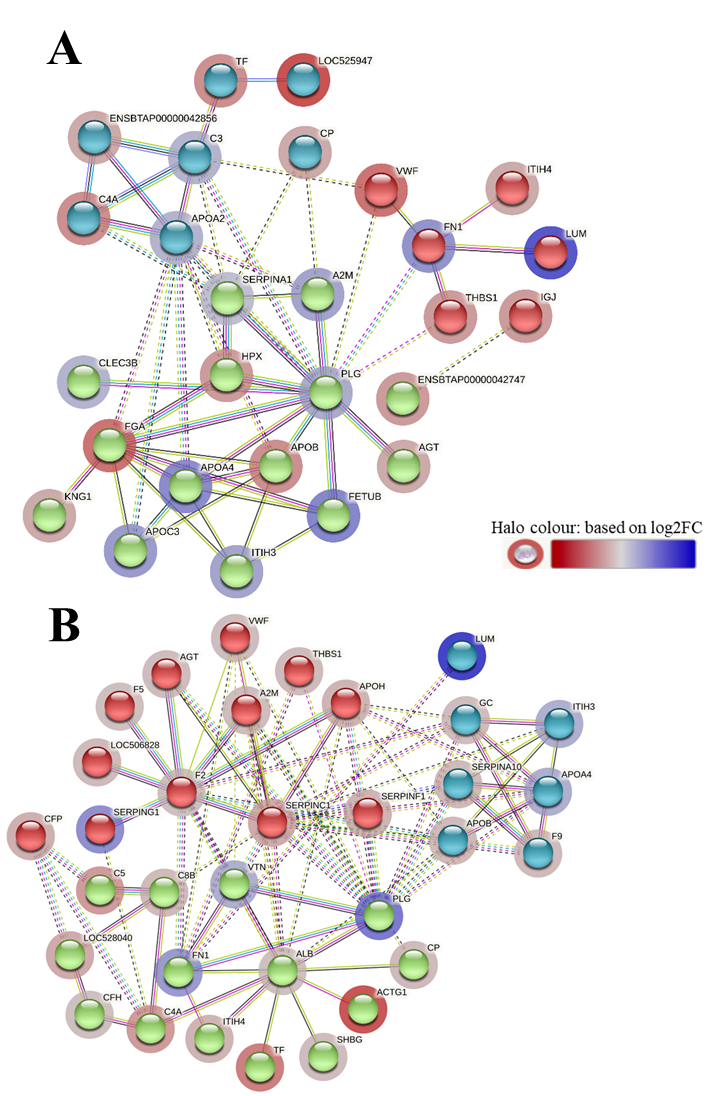
**

**Figure S1:** Functional protein-protein interaction among differentially abundant proteins in serum samples, **(A)** tick-susceptible (susceptible naïve vs S-105d PFI) and **(B)** resistant cattle resistant naïve vs R-105d PFI) in response to prolonged tick infestation. Each node represents an individual protein. *k*-mean clusters showing strong interactions are highlighted as “red”, “green” and “cyan blue” coloured nodes. The halo colour is based on the log_2_FC value of the proteins in the dataset.


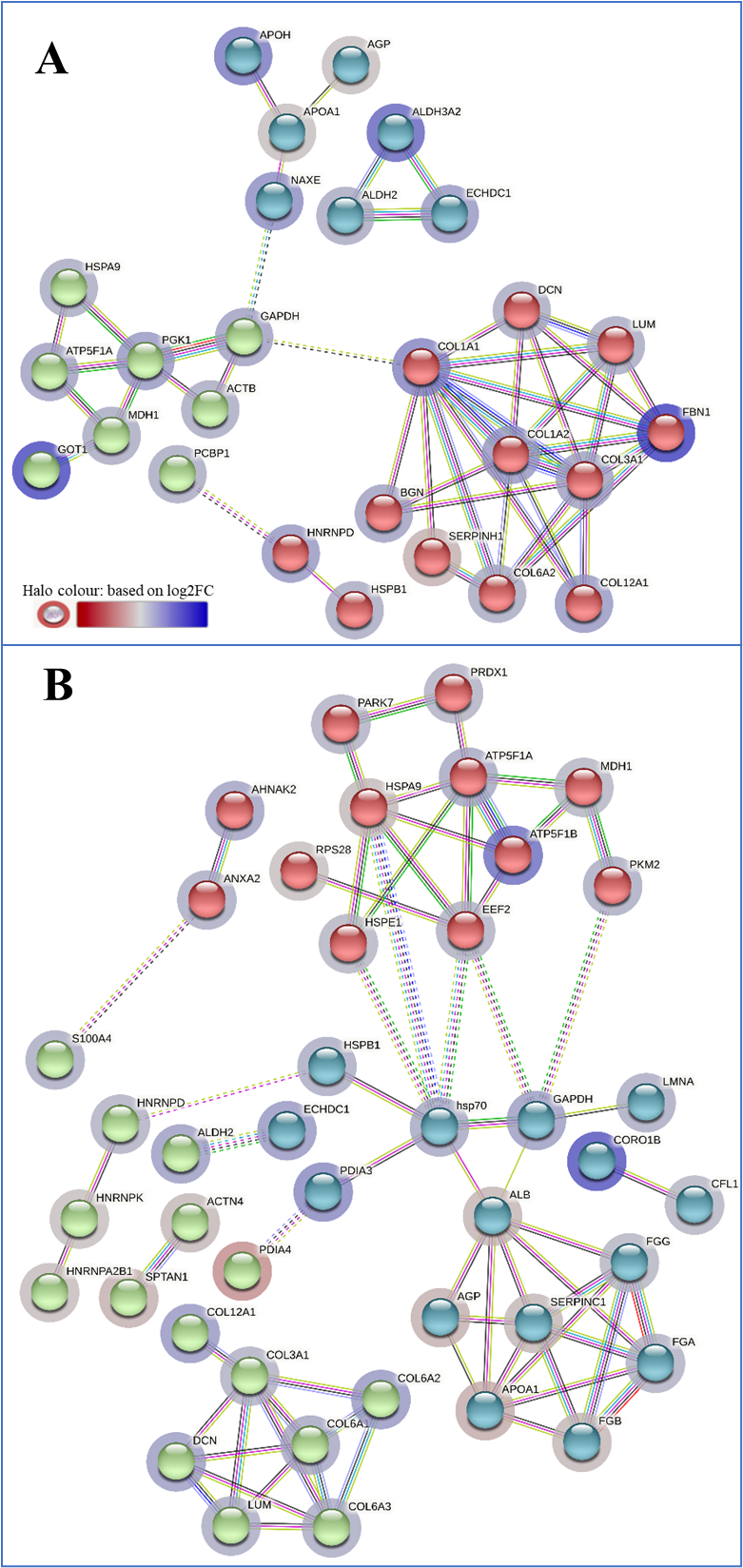


**Figure S2:** Functional protein-protein interaction among differentially abundant proteins in response to early tick infestation in skin samples. **(A)** tick-susceptible (susceptible naïve vs S-6hr PFI) and **(B)** resistant cattle (resistant naïve vs R-6hr PFI). Each node represents an individual protein. *k*-mean clusters showing strong interactions are highlighted as “red”, “green” and “cyan blue” coloured nodes. The halo colour is based on the log_2_FC value of the proteins in the dataset.


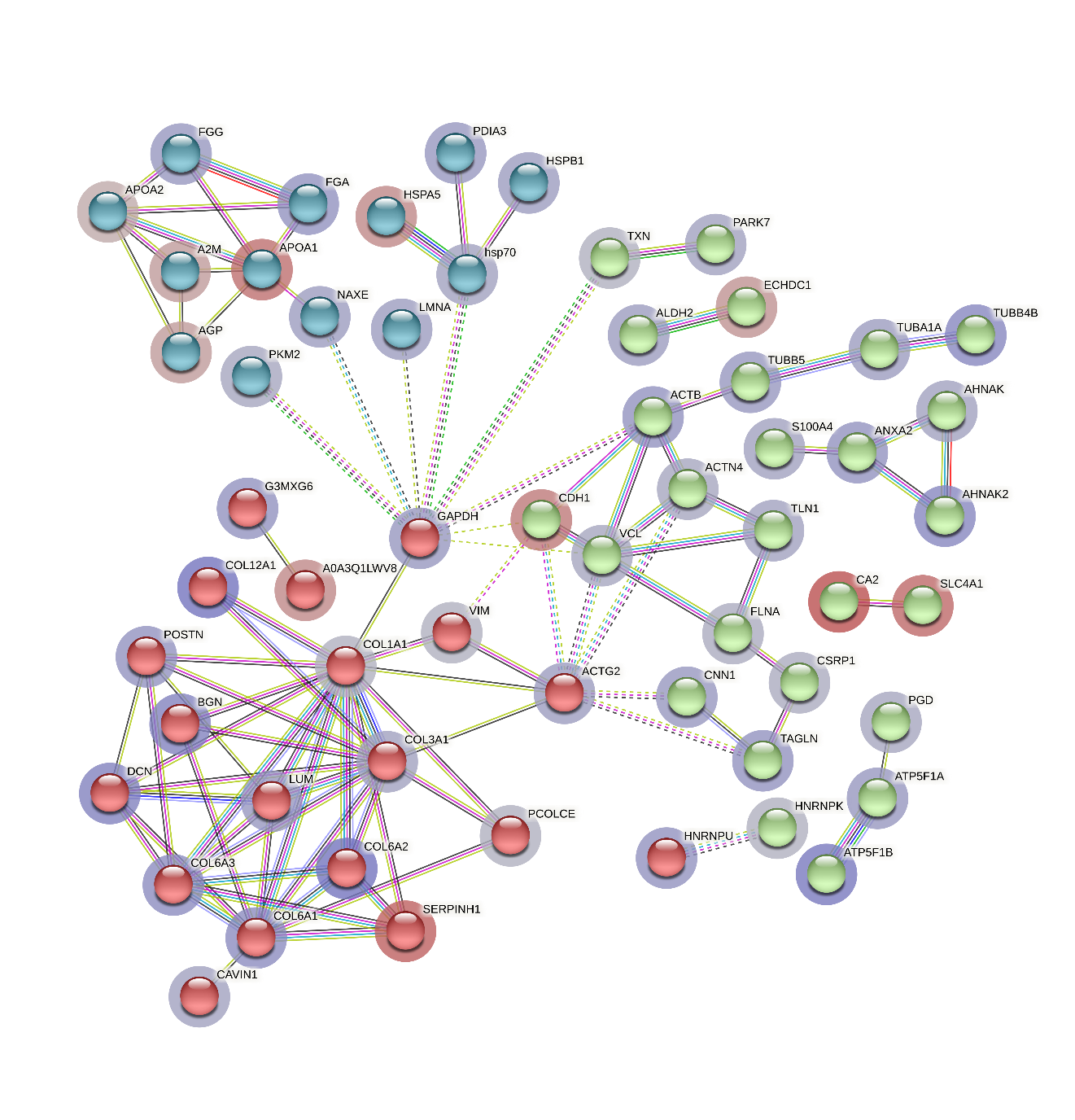

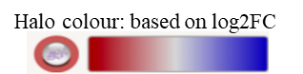


**Figure S3:** Functional protein-protein interaction among differentially abundant proteins in response to prolonged tick infestation in skin samples of resistant cattle (resistant naïve vs R-105d PFI). Each node represents an individual protein. *k*-mean clusters showing strong interactions are highlighted as “red”, “green” and “cyan blue” coloured nodes. The halo colour is based on the log_2_FC value of the proteins in the dataset.


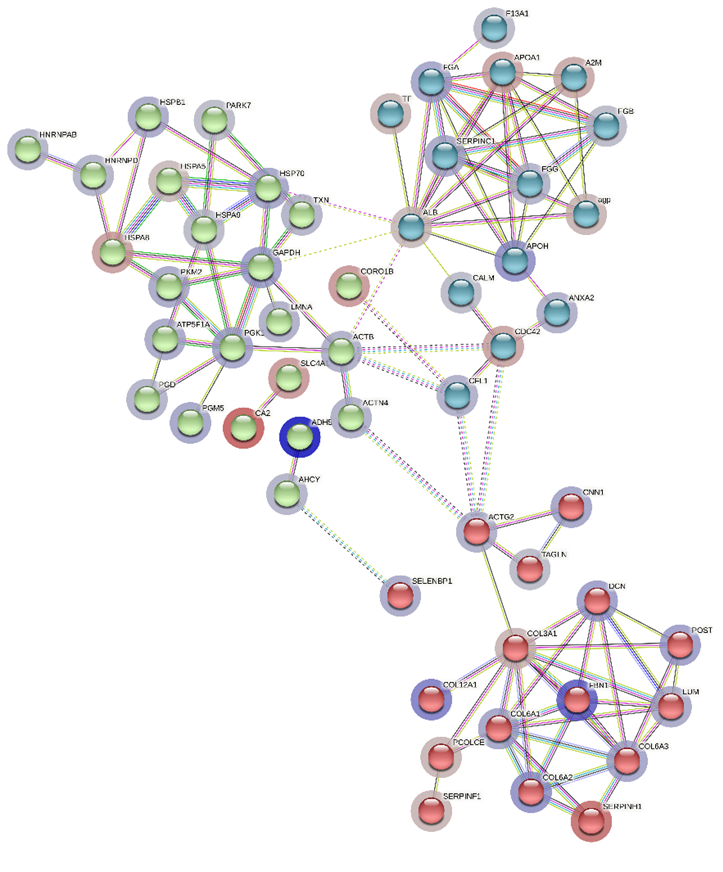

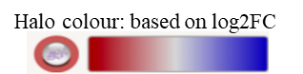


**Figure S4:** Functional protein-protein interaction among differentially abundant proteins in response to prolonged tick infestation in skin samples of tick-susceptible (susceptible naïve vs S-105d PFI) cattle. Each node represents an individual protein. *k*-mean clusters showing strong interactions are highlighted as “red”, “green” and “cyan blue” coloured nodes. The halo colour is based on the log_2_FC value of the proteins in the dataset.


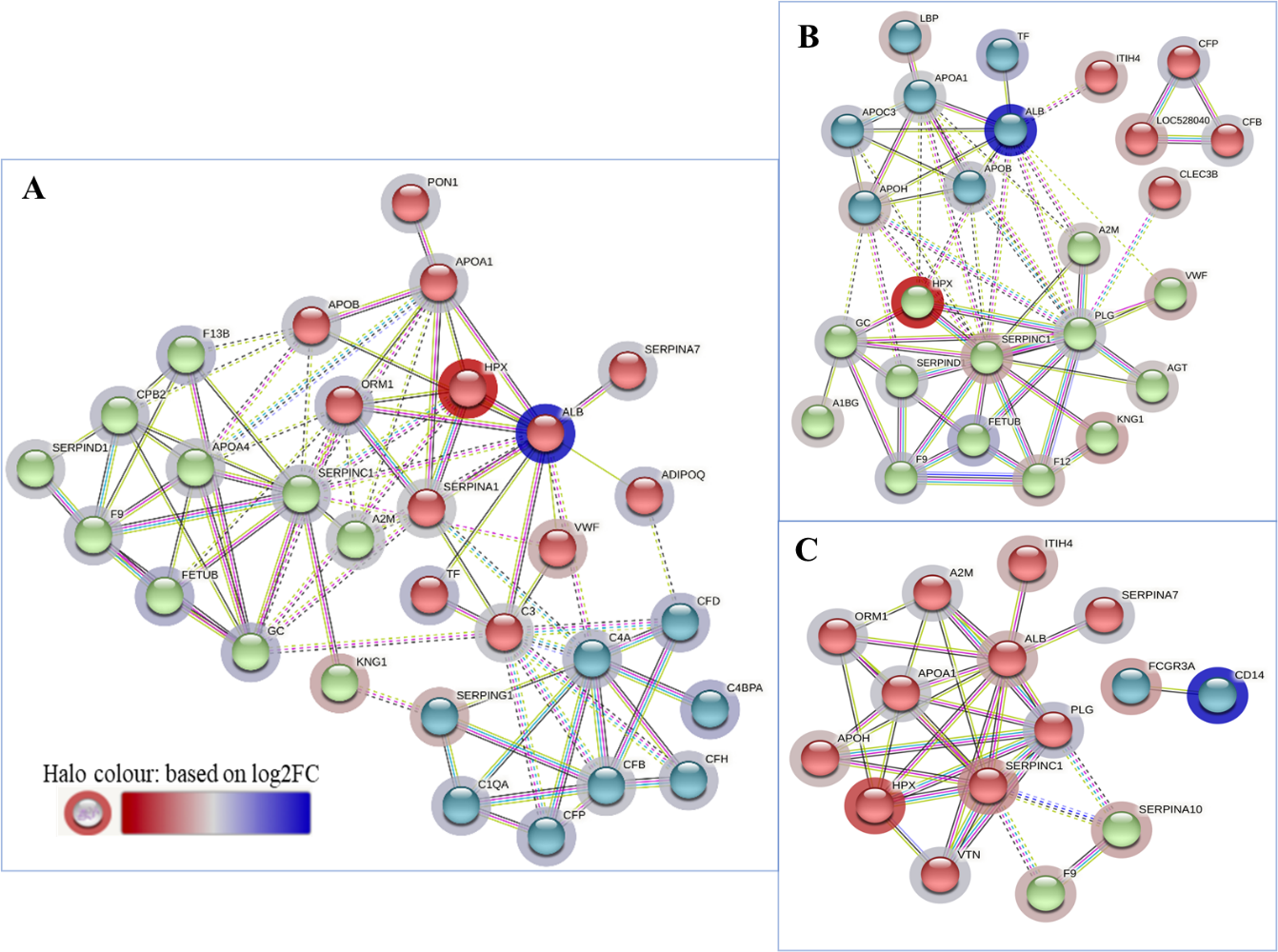
**Figure S5:** Functional protein-protein interaction networks among differentially abundant proteins in serum samples of tick-resistant cattle compared to the susceptible group **(A)** before infestation (resistant naïve vs susceptible naïve), **(B)** after early (R- 6h PFI vs S 6h PFI), and **(C)** prolonged tick infestation (R-105d PFI vs S-105d PFI). Each node represents an individual protein. *k*-mean clusters showing strong interactions are highlighted as “red”, “green”, and “cyan blue” coloured nodes. The halo colour is based on the log_2_FC value of the proteins in the dataset.


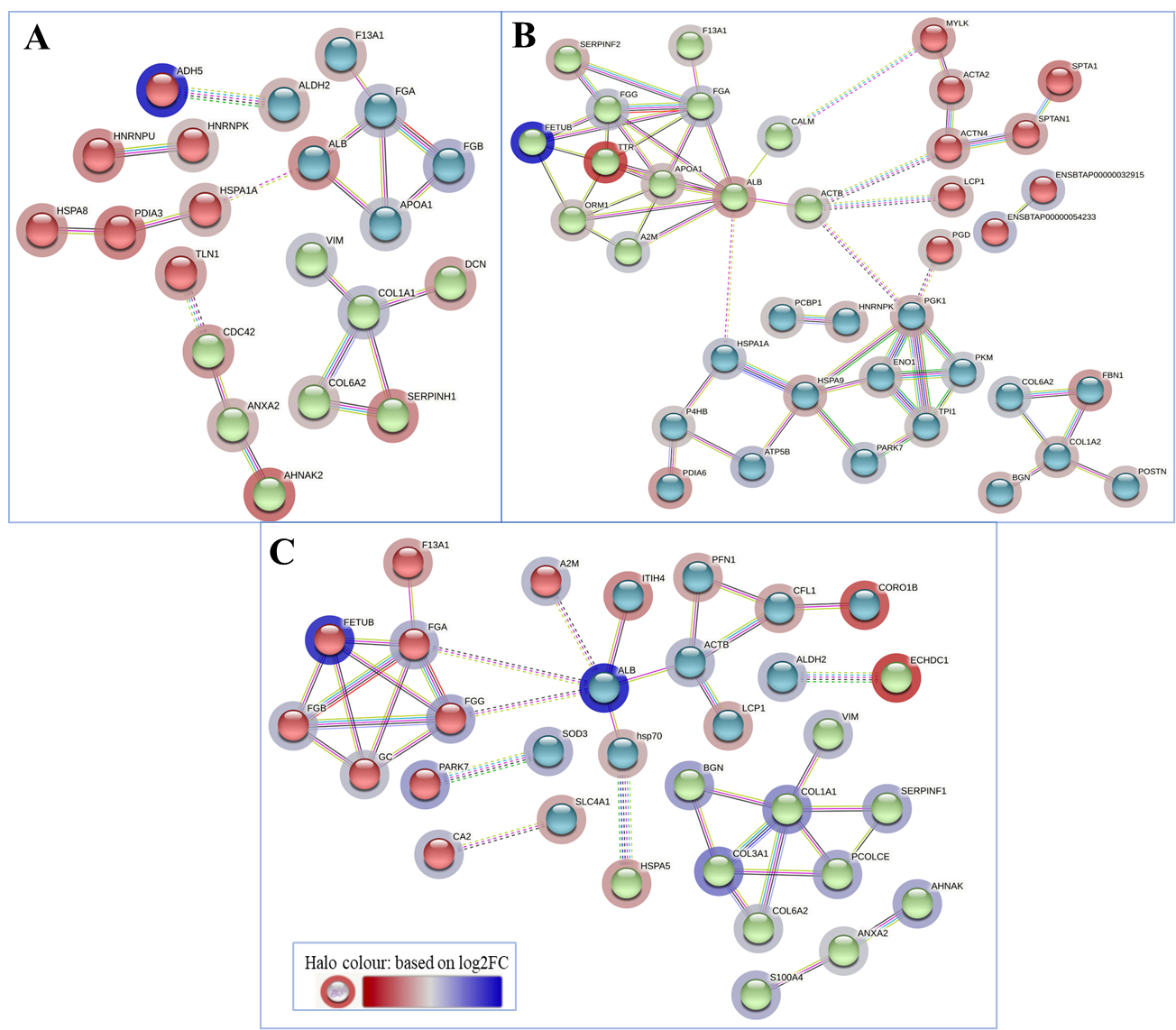
**Figure S6:** Functional protein-protein interaction networks among differentially abundant proteins in skin samples of tick-resistant cattle compared to the susceptible group **(A)** before infestation (resistant naïve vs susceptible naïve), **(B)** after early (R- 6h PFI vs S 6h PFI), and **(C)** prolonged tick infestation (R-105d PFI vs S-105d PFI). Each node represents an individual protein. *k*-mean clusters showing strong interactions are highlighted as “red”, “green”, and “cyan blue” coloured nodes. The halo colour is based on the log_2_FC value of the proteins in the dataset.


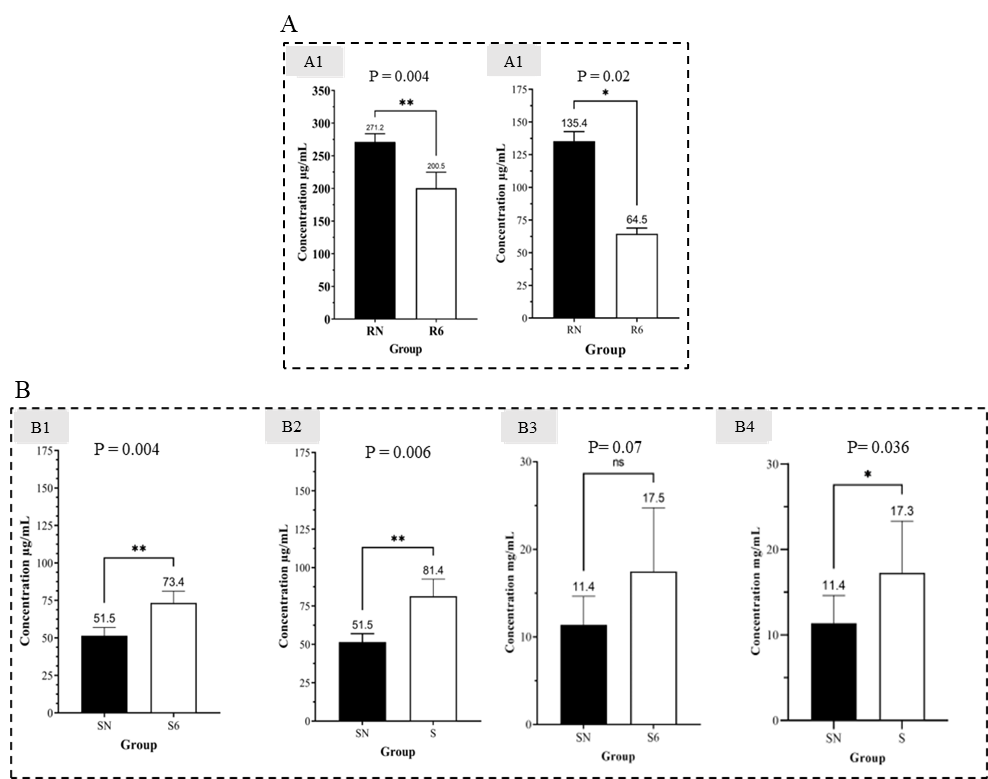
**Figure S7.** Bar graphs showing the concentrations of alpha-1-acid glycoprotein (AGP), conglutinin (CNG1) and immunoglobulin g (IgG) in sera of resistant and susceptible groups of cattle compared to their relevant baseline (naïve) samples measured by ELISA. **(A)** Concentration of AGP **(A-1)** and CNG1**(A-2)** in resistant naïve (RN) compared to resistant cattle after early tick exposure (RN vs R 6hr PFI). **(B)** Concentration of CNG1 in susceptible cattle compared to susceptible 6 hr PFI **(B-1)** and susceptible 105d PFI **(B-2)** samples. Concentration of IgG in susceptible cattle compared to susceptible 6 hr PFI **(B-3)** and susceptible 105d PFI **(B-4)** samples. Black bars represent naïve (resistant or susceptible) samples, and empty bars represent post-exposure samples (early or prolonged).
